# Meningeal lymphatics regulate radiotherapy efficacy through modulating anti-tumor immunity

**DOI:** 10.1038/s41422-022-00639-5

**Published:** 2022-03-17

**Authors:** Changping Zhou, Lu Ma, Han Xu, Yingqing Huo, Jincai Luo

**Affiliations:** Laboratory of Vascular Biology, Institute of Molecular Medicine, College of Future Technology, Beijing Key Laboratory of Cardiometabolic Molecular Medicine, Peking University, Beijing, China

**Keywords:** Tumour immunology, Radiotherapy

## Abstract

As a first-line treatment, radiotherapy (RT) is known to modulate the immune microenvironment of glioma, but it is unknown whether the meningeal lymphatic vessel (MLV)-cervical lymph node (CLN) network regulates the process or influences RT efficacy. Here, we show that the MLV-CLN network contributes to RT efficacy in brain tumors and mediates the RT-modulated anti-tumor immunity that is enhanced by vascular endothelial growth factor C (VEGF-C). Meningeal lymphatic dysfunction impaired tumor-derived dendritic cell (DC) trafficking and CD8^+^ T cell activation after RT, whereas tumors overexpressing VEGF-C with meningeal lymphatic expansion were highly sensitive to RT. Mechanistically, VEGF-C-driven modulation of RT-triggered anti-tumor immunity was attributed to C-C Motif Chemokine Ligand 21 (CCL21)-dependent DC trafficking and CD8^+^ T cell activation. Notably, delivery of VEGF-C mRNA significantly enhanced RT efficacy and anti-tumor immunity in brain tumors. These findings suggest an essential role of the MLV-CLN network in RT-triggered anti-tumor immunity, and highlight the potential of VEGF-C mRNA for brain tumor therapy.

## Introduction

Brain tumors, including primary and metastatic tumors, are among the most feared of all forms of cancers.^1,2^ In adults, glioblastomas, the most common primary brain tumors, cause nearly universal mortality and a median survival time of <21 months, despite surgical resection, targeted radiation therapy, and high-dose chemotherapy.^3^ Immune checkpoint inhibition and chimeric antigen receptor (CAR)-T cell therapy have been approved by US Food and Drug Administration (FDA) and clinically efficacious in several cancers, and other therapies such as cancer vaccines and dendritic cell (DC)-based vaccines have shown promise in clinical trials in many tumor types, but their efficacy thus far has been limited in glioma.^3,4^ Nevertheless, unsatisfactory clinical trials with immunotherapies indicate an improved immune response in gliomas after treatment.^1,5,6^ Interestingly, recent studies have demonstrated that neoadjuvant pembrolizumab therapy, which is one injection of an anti-programmed cell death protein 1 (PD-1) immune checkpoint antibody followed by surgical resection of the tumor in glioma patients, promoted survival as compared to adjuvant pembrolizumab, accompanied by improved anti-tumor immune responses, such as increased T cell infiltration and cytotoxic gene expression, and enhanced clonal expansion of T cells.^7–9^ These studies indicate that combination of immune and conventional therapies may be a potential strategy to improve clinical efficacy, which needs to be explored further.

Radiotherapy (RT), one of main conventional therapies for glioma and metastatic brain tumors, irreversibly damages tumor cell DNA and leads to apoptotic cell death, which is thought to be its major mode of action.^10^ Over the past decade, accumulating evidence has shown that local irradiation of solid tumors, including gliomas, has immunomodulatory effects.^11–14^ In a mouse model of intracranial glioma, stereotactic radiosurgery augmented an anti-tumor immune response against glioma, primarily mediated by expansion of the cytotoxic CD8^+^ T cell population.^15^ Further studies using a combination therapy strategy have demonstrated that RT contributes to a durable survival benefit for significant proportions of animals with glioma, likely through a T cell-dependent anti-tumor mechanism.^16^ These studies have opened new avenues to explore the role played by radiation in inducing anti-glioma immune responses and lay the basis of ongoing phase I/II trials for glioma patients using RT combination treatments.^17^ So far, however, the mechanisms underlying RT-induced anti-glioma immune responses are poorly understood. Especially, it remains unclear how RT modulates these T cell-dependent immune responses in gliomas and whether RT-triggered anti-tumor responses can be manipulated.

For a long time, the central nervous system (CNS) had been commonly accepted to be a system without classic lymphatic vessels. The rediscovery of meningeal lymphatic vessels (MLVs) in the dura mater has provided a new aspect of immunity in the CNS.^18^ MLVs possess some of the classic lymphatic functions such as drainage and immunoregulation.^18,19^ Like a bridge, MLVs make connections between the brain and cervical lymph nodes (CLNs) and are open to the trafficking of immune cells such as T cells^19^ and DCs.^20^ Vascular endothelial growth factor C (VEGF-C), a lymphangiogenesis factor, induces MLV expansion and then, via vascular endothelial growth factor receptor 3 (VEGFR3), promotes drainage of tracer from the CNS.^21,22^ MLVs are involved in many CNS diseases, including brain tumors,^20,23^ by means of immunomodulation.^19,21,22,24^ Our recent study demonstrated that the numbers of CD45^+^, CD68^+^, and CD3e^+^ cells were dramatically reduced in intracranial GL261 gliomas after dorsal MLVs were disrupted.^20^ In addition, the immunotherapy with anti-PD-1/CTLA-4 treatment increased in the total number of CD8^+^ T cells and CD8^+^ Ki67^+^ T cells in an MLV-dependent manner, consistent with previous results that immune checkpoint inhibition increased the number^25^ and proliferation (as measured by Ki67) of CD8^+^ T cells.^26^ Besides, this treatment also decreased the number of CD4^+^ Foxp3^+^ T_reg_ cells, which were shown to play an immune-suppressive role in an early study.^27^ Notably, the rate of IFNγ-producing CD8^+^ T cells was significantly increased in MLV-intact mice but not in MLV-defective mice after the treatment, which was correlated with the increase in the percentage of CD8^+^ Ki67^+^ T cells and the ratio of CD8^+^ Ki67^+^ T cells to CD4^+^ Foxp3^+^ T_reg_ cells. Almost simultaneously, another study demonstrated that MLV not only strengthens the inhibitory effect of immune checkpoint blockade but also enables the immunosurveillance of brain tumors.^23^ These studies suggest an important role of MLVs in the anti-glioma immune response induced by immunotherapy. Nevertheless, whether MLVs participate in RT-triggered immune modulation and tumor inhibition is unknown. In this study, we sought to evaluate the role of MLVs in the anti-tumor effect of RT in mouse models with intracranial GL261 glioma or experimentally metastasized Lewis lung carcinoma (LLC), and to gain insight into the involvement and mechanism of MLVs in RT-induced immune responses.

## Results

### The therapeutic effect of RT on glioma is dependent on intact MLVs and CLNs, which is related with anti-tumor immune responses

**Fig. 1:**
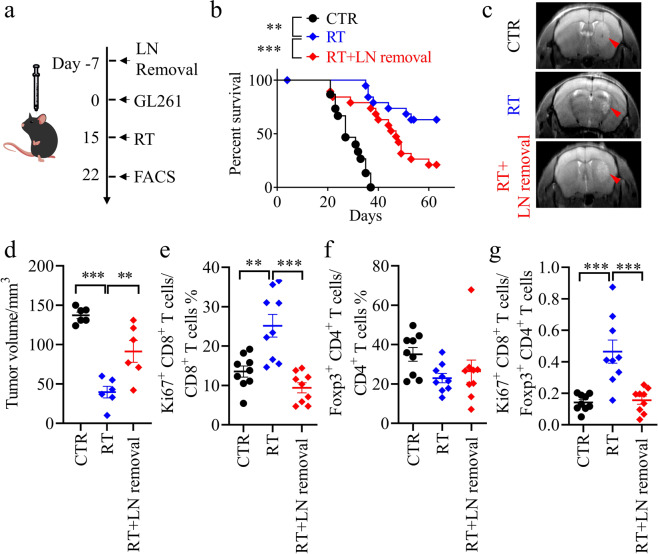
LN removal weakens the therapeutic effect of RT. **a** Scheme of the mouse GL261 glioma model with RT. **b** Survival of mice after striatal GL261 tumor injection, without treatment (CTR, *n* = 15), treated with RT alone (RT, *n* = 20), or treated with RT plus LN removal (RT + LN removal, *n* = 19). **c** Representative T2-weighted single brain slices from the CTR, RT, and RT + LN removal groups (triangles indicate tumors). **d** Tumor volume quantified from MRI images of the above groups on day 22 after inoculation (*n* = 6). **e**–**g** CD8^+^ Ki67^+^ T cells as percentages of the total CD8^+^ T cells (**e**), CD4^+^ Foxp3^+^ T cells as percentages of the total CD4^+^ T cells (**f**), and ratios of CD8^+^ Ki67^+^ T cells to CD4^+^ Foxp3^+^ T cells (**g**) in tumors from the above groups on day 22 after inoculation (*n* = 9). Data are presented as means ± SEM. *P < 0.05, **P < 0.01, ***P < 0.001; log-rank (Mantel–Cox) test (**b**); one-way ANOVA (**d**–**g**). Data are from at least three (**b**, **e**–**g**) or two (**c**, **d**) independent experiments.

Tumor-draining lymph nodes (TDLNs) are key sites for tumor antigen presentation and T cell priming.^28^ The CLNs collect lymph from MLVs^18,29^ and serve as the primary draining lymph nodes (LNs) of brain tumors.^20,23^ To define the contribution of MLVs and CLNs to the treatment of brain tumors by RT and to find out how interruption of this system might affect anti-tumor immunity, we first surgically resected the CLNs of C57BL/6 mice, which then were given an injection of syngeneic GL261 glioma cells into the striatum before RT (Fig. 1a). We found that RT significantly prolonged the survival of mice bearing gliomas and decreased the tumor size (Fig. 1b–d). In contrast, this therapeutic effect was impaired when CLNs were removed (Fig. 1b–d). MLV-CLN network is known to be involved in anti-tumor immunity via promotion of the infiltration and activation of CD8^+^ T cells in gliomas.^20,23^ Consistently, the percentage of CD8^+^ Ki67^+^ T cells and the ratio of CD8^+^ Ki67^+^ T cells to CD4^+^ Foxp3^+^ T_reg_ cells in gliomas were increased after RT, but decreased when CLNs were removed (Fig. 1e–g; Supplementary information, Fig. S2a, b). These data suggested that CLNs, at least in part, contribute to the treatment of gliomas by RT, possibly through mediating the activation and infiltration of CD8^+^ T cells into the tumors. Indeed, RT increased the percentage of CD8^+^ Ki67^+^ T cells and the ratio of CD8^+^ Ki67^+^ T cells to T_reg_ cells in the CLNs of mice with gliomas (Supplementary information, Fig. S3a–e). Besides, RT had little effect on tumor-associated macrophages and CD8^+^ TCF7^+^ T cells (Supplementary information, Fig. S3f, g).

**Fig. 2:**
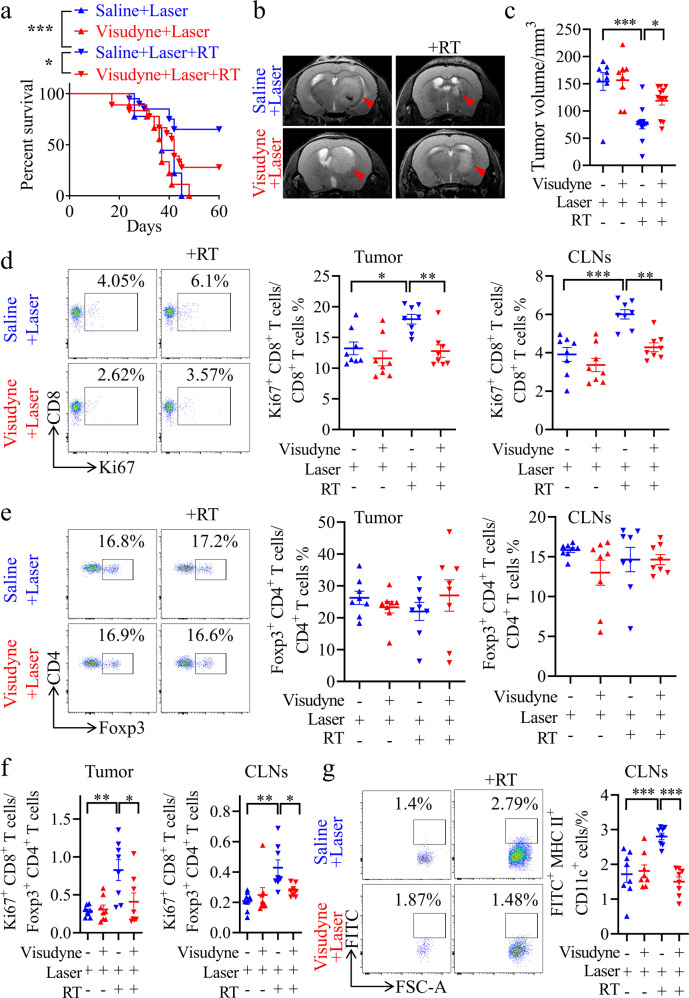
MLV ablation weakens the therapeutic effect of RT. **a** Survival of mice with striatal GL261 tumor injection with or without RT, saline plus laser treatment (Saline + Laser, *n* = 9; Saline + Laser + RT, *n* = 20), or Visudyne plus laser treatment (Visudyne + Laser, *n* = 8; Visudyne + Laser + RT, *n* = 18). **b** Representative T2-weighted single brain slices from the above groups (triangles indicate tumors). **c** Tumor volume quantified from MRI images of the above groups on day 22 after inoculation (Saline + Laser, *n* = 8; Saline + Laser + RT, *n* = 12; Visudyne + Laser, *n* = 8; Visudyne + Laser + RT, *n* = 12). **d**, **e** Representative flow cytometry plots of CD8^+^ Ki67^+^ T cells as percentages of the total CD8^+^ T cells (**d**), and CD4^+^ Foxp3^+^ T cells as percentages of the total CD4^+^ T cells (**e**) in CLNs (left) and quantification (right) in tumors and CLNs from the above groups on day 22 after inoculation (*n* = 8). **f** Ratios of CD8^+^ Ki67^+^ T cells to CD4^+^ Foxp3^+^ T cells in tumors and CLNs from the above groups (*n* = 8). **g** Left panel, representative flow cytometry dot plots of DC trafficking from GL261 tumors to CLNs shown by the quantity of CD11c^+^ MHCII^+^ FITC^+^ cells (FITC^+^ DCs) in the CLNs 24 h after intratumoral injection of FITC-labeled latex beads. Right panel, quantification of FITC^+^ DCs as a fraction of all MHC II^+^ CD11c^+^ cells in CLNs (*n* = 8). Data are presented as means ± SEM. *P < 0.05, **P < 0.01, ***P < 0.001; log-rank (Mantel–Cox) test (**a**); one-way ANOVA (**c**–**g**). Data are from at least three (**a**, **d**–**g**) or two (**b**, **c**) independent experiments.

Then, we evaluated the role of MLVs in the infiltration and activation of CD8^+^ T cells into tumor tissue during RT to treat gliomas. GL261 cells were injected into the striatum of MLV-defective mice (Supplementary information, Fig. S4a, b), which were established using a method based on the photochemical effect of Visudyne under laser exposure.^19,20^ As shown in Fig. 2a–c, MLV deficiency shortened the survival of mice with gliomas, and increased the size of tumors after RT. This result was confirmed using another MLV-defective mouse model (Supplementary information, Fig. S5a–c), in which Prox1-CreRT2^+/–^ mice were bred with VEGFR3-flox^+/+^ mice (Prox1-CreRT2^–/–^;VEGFR3-flox^+/+^ mice as controls) as previously described.^30^ Consistent with a previous study,^31^ we observed that RT could increase the total CD8^+^ T cells in tumor in an MLV-dependent manner (Supplementary information, Fig. S4c, d). As expected, MLV deficiency decreased the percentage of CD8^+^ Ki67^+^ T cells and the ratio of CD8^+^ Ki67^+^ T cells to T_reg_ cells in both tumors and CLNs after RT (Fig. 2d–f). These results suggested a potential role of MLVs in RT-induced anti-tumor immunity.

Antigen-presenting cells (APCs), which carry tumor antigens, traffic to TDLNs through lymphatic vessels. DCs are the most important APCs in anti-glioma immunity priming.^32^ To determine whether MLVs activate CD8^+^ T cells by facilitating DC trafficking, we then quantified DC trafficking after RT by intratumoral injection of 0.5 μm FITC-labeled beads, which are too large to flow into MLVs and instead must be taken up by DCs in the tumor before being transported to the CLNs. As expected, CD11c^+^ MHCII^+^ FITC^+^ cells (FITC^+^ DCs) were increased in the CLNs after RT compared with control mice; but the percentage of these cells was dramatically reduced in MLV-defective mice compared with MLV-intact mice (Fig. 2g). Besides, RT also increased CD11c^+^ MHCII^+^ cells (total DCs) in the CLNs, which was dependent on intact MLV (Supplementary information, Fig. S4e). These results suggested that MLVs are the key route for the increased DC trafficking during RT. MLV ablation may impair the RT-induced DC trafficking and lead to the failure of T cell priming in CLNs.

The above results demonstrated the potential role of MLVs and CLNs in RT-induced anti-tumor immunity.

### VEGF-C level is strongly correlated with anti-tumor immune responses in glioma patients after RT

**Fig. 3:**
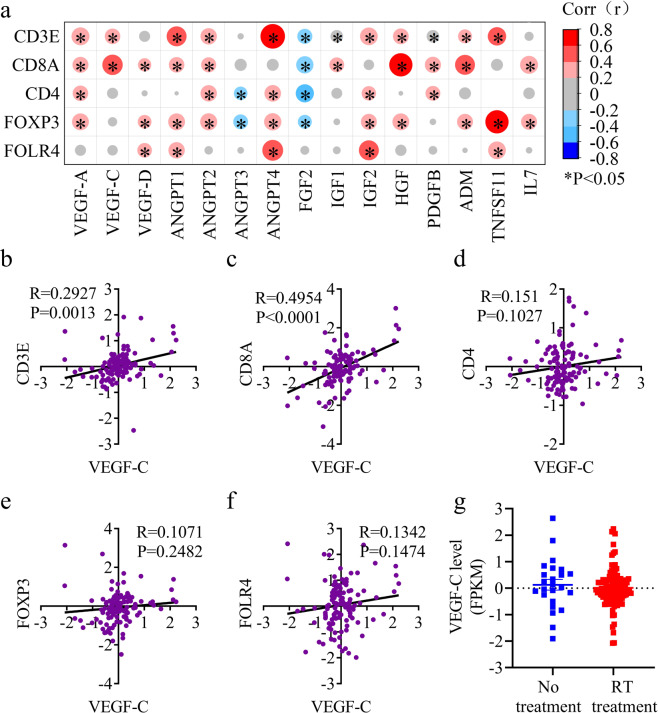
VEGF-C levels are correlated with T cell infiltration in glioma patients after RT. The data of glioma patients with RT were collected from the CGGA database, and the correlation was expressed by Pearson correlation coefficient (*R* value). **a** Correlation of lymphangiogenesis factors and T cell infiltration (measured by the expression of CD8A and CD4) in glioma patients with RT alone (data from the mRNA-array_301 database of the CCGA; *n* = 118). **b**–**f** Correlation of the expressions of VEGF-C and the T cell marker CD3E (**b**), the CD8^+^ T cell marker CD8A (**c**), and the T_reg_ markers CD4 (**d**), FOXP3 (**e**) and FOLR4 (**f**) in glioma patients with RT alone (data from the mRNA-array_301 database of the CCGA; *n* = 118). **g** VEGF-C expression (fragments per kilobase transcriptome per million fragments; FPKM) in glioma patients with no treatment or RT alone (data from the mRNA-array_301 database of the CCGA; no treatment, *n* = 24; RT alone, *n* = 118). *P* values are Pearson’s correlations.

Given the importance of the MLV-CLN system for RT, we hypothesized that enhanced meningeal lymphangiogenesis would potentiate the RT-induced anti-tumor immunity. We thus investigated the correlation between the gene expression of lymphangiogenesis factors and immune cell infiltration into the gliomas of patients after RT treatment by analyzing the mRNA-array_301 database of the Chinese Glioma Genome Atlas (CCGA).^33,34^ While hepatocyte growth factor (HGF) and adrenomedullin (ADM), among 15 well-known lymphangiogenesis factors,^35,36^ displayed correlations with both CD8^+^ T cell number and T_reg_ cell number (as measured by FOXP3^37^ and FOLR4^38^), *VEGF-C* appeared to be a unique gene that was highly correlated with CD8^+^ T cell number but had no correlation with T_reg_ cell number (Fig. 3a). In detail, the VEGF-C level was strongly correlated with the infiltration of T cells, as measured by the expression of the T cell marker CD3E (Fig. 3b). More importantly, the VEGF-C level was correlated with the expression of CD8^+^ T cell marker CD8A (Fig. 3c), while the correlation between the VEGF-C level and CD4/FOXP3/FOLR4 was weak (Fig. 3d–f), confirming the correlation between VEGF-C expression and CD8^+^ T cell infiltration. Similar correlations were found in the Gene Expression Omnibus database (GSE42669)^39^ (Supplementary information, Fig. S6a–d). Together, these data showed a correlation between VEGF-C expression and CD8^+^ T cell infiltration into human gliomas after RT, even though VEGF-C expression did not significantly differ in glioma patients with no treatment or RT treatment alone (Fig. 3g). Collectively, these results suggest that VEGF-C levels are correlated with T cell infiltration in glioma patients after RT.

### VEGF-C-overexpressing gliomas are highly sensitive to RT

**Fig. 4:**
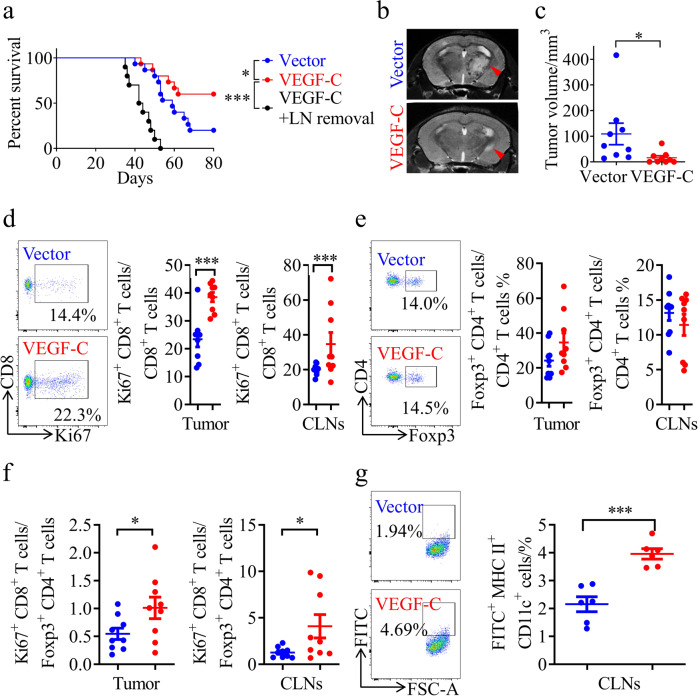
Tumor-derived VEGF-C improves the therapeutic effect of RT. **a** Survival of mice with striatal vector-GL261 or VEGF-C-GL261 tumor injection treated with RT (vector, *n* = 18; VEGF-C, *n* = 15; VEGF-C + LN removal, *n* = 10). **b** Representative T2-weighted single brain slices from mice with striatal vector-GL261 or VEGF-C-GL261 tumor injection treated with RT (triangles indicate tumors). **c** Tumor volume quantified from MRI images on day 22 after inoculation of mice with striatal vector-GL261 or VEGF-C-GL261 tumor injection treated with RT (*n* = 9). **d**, **e** Representative flow cytometry plots of CD8^+^ Ki67^+^ T cells as percentages of the total CD8^+^ T cells (**d**), and CD4^+^ Foxp3^+^ T cells as percentages of the total CD4^+^ T cells (**e**) in CLNs (left) and quantification (right) in tumors and CLNs from mice with striatal vector-GL261 or VEGF-C-GL261 tumor injection treated with RT on day 22 after inoculation (*n* = 9). **f** Ratios of CD8^+^ Ki67^+^ T cells to CD4^+^ Foxp3^+^ T cells in tumors and CLNs from mice with striatal vector-GL261 or VEGF-C-GL261 tumor injection treated with RT (*n* = 9). **g** Left panel, representative flow cytometry dot plots of DC trafficking from GL261 tumors to CLNs of mice with striatal vector-GL261 or VEGF-C-GL261 tumor injection treated with RT shown by the quantity of FITC^+^ DCs in the CLNs 24 h after intratumoral injection of FITC-labeled latex beads. Right panel, quantification of FITC^+^ DCs as a fraction of all MHC II^+^ CD11c^+^ cells in the CLNs of mice with striatal vector-GL261 or VEGF-C-GL261 tumor injection treated with RT (*n* = 6). Data are presented as means ± SEM. *P < 0.05, **P < 0.01, ***P < 0.001; log-rank (Mantel–Cox) test (**a**); Student’s *t*-test (**c**–**g**). Data are from at least three (**a**, **d**–**g**) or two (**b**, **c**) independent experiments.

Thus, we further investigated the correlations among the VEGF-C level, radiosensitivity, and immune cell infiltration in mouse gliomas overexpressing VEGF-C or harboring the vector control. We found that the VEGF-C overexpression group survived longer than the vector group after RT (Fig. 4a). Consistent with this, mice bearing tumors overexpressing VEGF-C displayed decreased tumor volumes compared to those bearing tumors with the vector control after RT (Fig. 4b, c). The radiosensitivity of VEGF-C-overexpressing gliomas was abolished after LN removal (Fig. 4a), indicating that VEGF-C promotes RT efficacy in an MLV-CLN system-dependent manner. In addition, we observed that VEGF-C overexpression in tumors increased the percentage of CD8^+^ Ki67^+^ T cells and the ratio of CD8^+^ Ki67^+^ T cells to T_reg_ cells in both tumors and CLNs (Fig. 4d–f), and increased the total CD8^+^ T cells in CLNs (Supplementary information, Fig. S7a, b). Furthermore, using an assay by injecting FITC-labeled beads into tumors, we found that the percentage of FITC^+^ DCs increased in the CLNs of mice with VEGF-C-overexpressing gliomas after RT (Fig. 4g). Besides, VEGF-C overexpression in tumors also increased the percentage of total DCs after RT (Supplementary information, Fig. S7c). These results suggest that VEGF-C enhances anti-tumor immunity probably via the promotion of DC trafficking.

### VEGF-C enhances the therapeutic effect of RT on gliomas through a C-C Motif Chemokine Ligand 21 (CCL21)-dependent mechanism

**Fig. 5:**
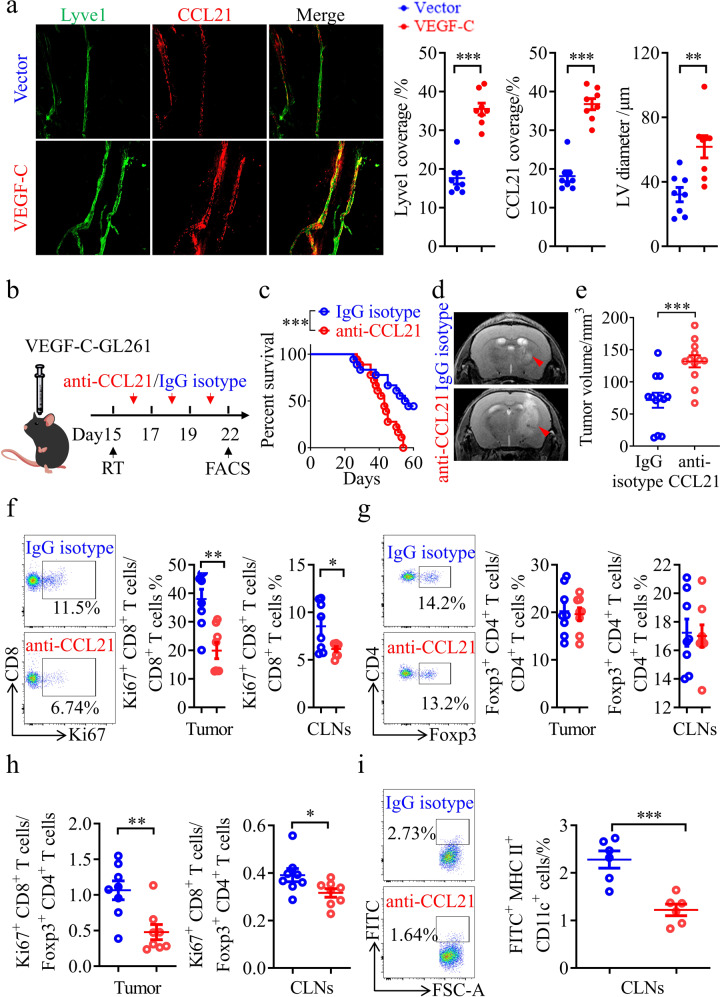
CCL21 blockade abolishes the RT efficiency and anti-tumor immunity enhanced by VEGF-C overexpression. **a** Left panels, Lyve1 and CCL21 staining of MLVs in mice bearing vector-GL261 or VEGF-C-GL261 tumors in the striatum with RT. Right panels, quantification of the coverage percentage of Lyve1 or CCL21, and the LV diameter (*n* = 8). **b** Monitoring and treatment scheme. CCL21 was blocked on days 16, 18, and 20 after inoculation. **c** Survival of mice with striatal VEGF-C-GL261 tumor injection treated with RT in the presence or absence of anti-CCL21 antibody (*n* = 18). **d** Representative T2-weighted single brain slices from mice in IgG isotype or anti-CCL21 groups (triangles indicate tumors). **e** Tumor volume quantified from MRI images on day 22 after inoculation of mice from IgG isotype or anti-CCL21 groups (*n* = 12). **f**, **g** Representative flow cytometry plots of CD8^+^ Ki67^+^ T cells as percentages of the total CD8^+^ T cells (**f**), and CD4^+^ Foxp3^+^ T cells as percentages of the total CD4^+^ T cells (**g**) in CLNs (left) and quantification (right) in tumors and CLNs from IgG isotype or anti-CCL21 groups on day 22 after inoculation (*n* = 8). **h** Ratios of CD8^+^ Ki67^+^ T cells to CD4^+^ Foxp3^+^ T cells in tumors and CLNs from IgG isotype or anti-CCL21 groups (*n* = 8). **i** Left panel, representative flow cytometry dot plots of DC trafficking from GL261 tumors to CLNs of mice in IgG isotype or anti-CCL21 groups shown by the quantity of CD11c^+^ MHCII^+^ FITC^+^ cells in the CLNs 24 h after intratumoral injection of FITC-labeled latex beads. Right panel, quantification of FITC^+^ DCs as a fraction of all MHC II^+^ CD11c^+^ cells in the CLNs of mice from the IgG isotype or anti-CCL21 group (*n* = 6). Data are presented as means ± SEM. *P < 0.05, **P < 0.01, ***P < 0.001; log-rank (Mantel–Cox) test (**b**); Student’s *t*-test (**a**, **c**–**i**). Data are from at least three (**a**, **c**, **f**–**i**) or two (**d**, **e**) independent experiments.

To explore how VEGF-C facilitates DC trafficking after RT, we assessed the differences in MLVs between the VEGF-C and vector control groups. As expected, VEGF-C overexpression in tumors promoted MLV expansion, as evidenced by larger MLV diameters and greater MLV coverage in the VEGF-C group (Fig. 5a). More importantly, CCL21 expression was higher in the lymphatic endothelial cells (LECs) of MLVs after VEGF-C overexpression (Fig. 5a). CCL21 is the most important LEC-derived chemokine for DC recruitment.^40^ To determine whether the CCL21 pathway mediates the facilitation of anti-tumor immunity by VEGF-C overexpression, IgG isotype or anti-CCL21 antibodies were each administered to mice bearing gliomas that overexpressed VEGF-C. RT treatment was followed by anti-CCL21 antibody administration a few days later to avoid confounding effects (Fig. 5b). The data showed that anti-CCL21 antibodies reduced the survival time after RT of mice bearing gliomas overexpressing VEGF-C (Fig. 5c). Besides, the tumor volume was significantly larger in the anti-CCL21 treatment group than in the IgG isotype group (Fig. 5d, e). We further analyzed the T cell changes in tumors and CLNs from these groups, and found that the percentage of CD8^+^ Ki67^+^ T cells was dramatically decreased in tumors and CLNs after anti-CCL21 treatment (Fig. 5f; Supplementary information, Fig. S8a). Although the percentage of CD4^+^ Foxp3^+^ T_reg_ cells showed no significant changes (Fig. 5g; Supplementary information, Fig. S8b), the ratio of CD8^+^ Ki67^+^ T cells to CD4^+^ Foxp3^+^ T_reg_ cells in tumors significantly differed between the anti-CCL21 and IgG isotype groups (Fig. 5h). Furthermore, anti-CCL21 treatment decreased the FITC^+^ DCs in the CLNs after RT, compared to IgG isotype treatment (Fig. 5i). Collectively, CCL21 blockade reversed the increased efficacy of RT treatment in mice bearing tumors overexpressing VEGF-C, suggesting that VEGF-C potentiates RT and this is dependent on the CCL21 pathway.

### VEGF-C mRNA robustly increases the therapeutic effect of RT on glioma

**Fig. 6:**
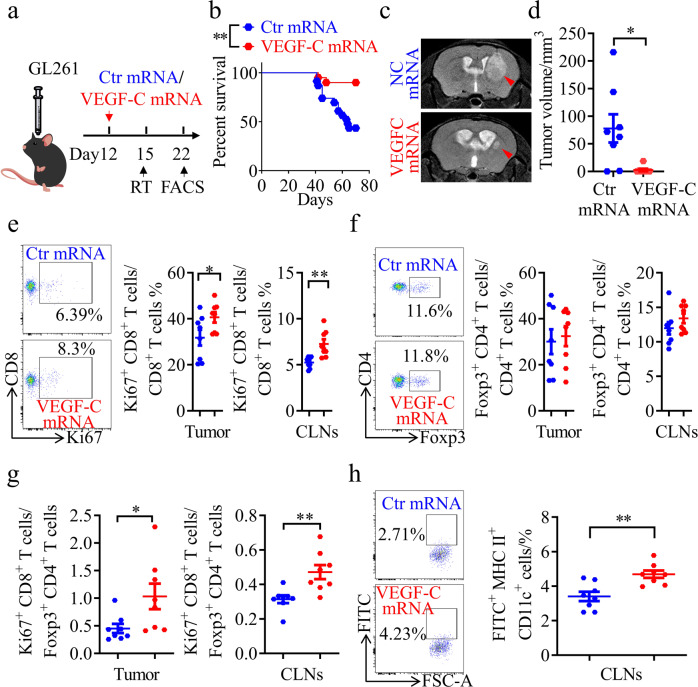
VEGF-C mRNA notably enhances the efficiency of RT. **a** Monitoring and treatment scheme. mRNA was injected on day 12 after inoculation. **b** Survival of mice with striatal GL261 tumor injection treated with RT in the presence or absence of VEGF-C mRNA (Ctr mRNA, *n* = 23; VEGF-C mRNA, *n* = 20). **c** Representative T2-weighted single brain slices from mice in the Ctr mRNA or VEGF-C mRNA groups (triangles indicate tumors). **d** Tumor volume quantified from MRI images on day 22 after inoculation of mice from Ctr mRNA or VEGF-C mRNA groups (*n* = 8). **e**, **f** Representative flow cytometry plots of CD8^+^ Ki67^+^ T cells as percentages of the total CD8^+^ T cells (**e**), and CD4^+^ Foxp3^+^ T cells as percentages of the total CD4^+^ T cells (**f**) in CLNs (left) and quantification (right) in tumors and CLNs from Ctr mRNA or VEGF-C mRNA groups on day 22 after inoculation (*n* = 8). **g** Ratios of CD8^+^ Ki67^+^ T cells to CD4^+^ Foxp3^+^ T cells in tumors and CLNs from Ctr mRNA or VEGF-C mRNA groups (*n* = 8). **h** Left panel, representative flow cytometry dot plots of DC trafficking from GL261 tumors to CLNs of mice in the above groups shown by the quantity of CD11c^+^ MHCII^+^ FITC^+^ cells in the CLNs 24 h after intratumoral injection of FITC-labeled latex beads. Right panel, quantification of FITC^+^ DCs as a fraction of all MHC II^+^ CD11c^+^ cells in the CLNs of Ctr mRNA or VEGF-C mRNA groups (*n* = 8). Data are presented as means ± SEM. *P < 0.05, **P < 0.01; log-rank (Mantel–Cox) test (**b**); Student’s *t*-test (**d**–**h**). Data are from at least three (**b**, **e**–**h**) or two (**c**, **d**) independent experiments.

VEGF-C mRNA has been shown to be a potential tool for anti-glioma immunity enhancement in mice.^23^ In this study, we designed an engineered mRNA of VEGF-C comprising an activated form of VEGF-C^41^ and immunoglobulin kappa (Igκ),^42^ an artificial signal peptide at the N-terminus of the activated form of VEGF-C (Supplementary information, Fig. S9a). Then, we employed delivery of this VEGF-C mRNA as a potential strategy to enhance the efficacy of RT on glioma and metastatic brain cancer. After VEGF-C mRNA injection via the posterior cistern, an increase in the activated form of VEGF-C protein was detected in the cerebrospinal fluid (CSF) (Supplementary information, Fig. S9b, c). VEGF-C mRNA induced the remodeling of MLVs, and promoted the expression of CCL21 (Supplementary information, Fig. S9d). Most importantly, the data showed that mice treated with VEGF-C mRNA survived much longer than those treated with control mRNA after RT (Fig. 6a, b). Consistent with this, the VEGF-C mRNA group displayed a decreased tumor volume after RT (Fig. 6c, d). Furthermore, flow cytometry showed that VEGF-C mRNA increased the total CD8^+^ T cells, the percentage of CD8^+^ Ki67^+^ T cells and the ratio of CD8^+^ Ki67^+^ T cells to CD4^+^ Foxp3^+^ T_reg_ cells in both tumors and CLNs (Fig. 6e–g; Supplementary information, Fig. S9e, f). The above results suggested that VEGF-C mRNA promotes T cell priming in CLNs and CD8^+^ T cell infiltration into brain tumors. Furthermore, increased FITC^+^ DCs were detected in the CLNs from the VEGF-C mRNA group after RT (Fig. 6h). Thus, VEGF-C mRNA may be a potential therapeutic drug for RT enhancement.

### VEGF-C mRNA also enhances the therapeutic effect of RT on metastatic brain tumor

To expand the above findings, we also investigated the contribution of CLNs to the treatment of metastatic brain tumors by RT and the effect of VEGF-C overexpression on RT-triggered anti-tumor responses, using an experimental brain metastasis model in which syngeneic LLC cells were injected into the striatum of C57BL/6 mice. RT increased the ratio of CD8^+^ Ki67^+^ T cells to T_reg_ cells in the CLNs of mice with intracranial metastases from lung cancer (Supplementary information, Fig. S10a–e). As shown in Supplementary information, Fig. S10f–i, RT significantly prolonged the survival of mice with intracranial LLC and decreased tumor size, but this therapeutic effect was compromised when CLNs were removed (Supplementary information, Fig. S10f–i). Compared to the vector group, the VEGF-C overexpression group survived longer and had smaller tumors after RT (Supplementary information, Fig. S11a–c). Consistent with this, the RT-triggered anti-tumor response in intracranial LLC, as measured by the number and proliferation of CD8^+^ T cells, was stronger in the VEGF-C overexpression group than in the vector group (Supplementary information, Fig. S11d–i). Strikingly, mice with intracranial LLC treated with VEGF-C mRNA survived longer and showed decreased tumor volume compared to those treated with control mRNA after RT (Supplementary information, Fig. S12a–d), which might be due to increased DC trafficking and CD8^+^ T cell activation (Supplementary information, Fig. S12e–j). These data showed that VEGF-C also enhances RT-triggered anti-tumor immune responses in metastatic brain tumors.

### VEGF-C mRNA significantly promotes T cell clonality and cytokine expression in glioma after RT

**Fig. 7:**
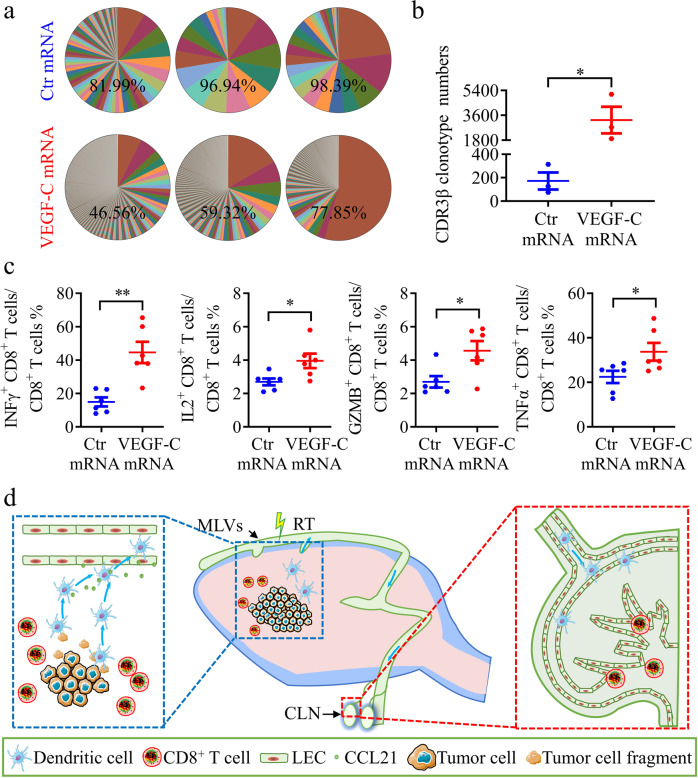
VEGF-C mRNA significantly promotes T cell function in glioma after RT. **a** Percentage of the top 50 most frequent clones of CD3β in tumors from Ctr mRNA or VEGF-C mRNA groups on day 22 after inoculation (*n* = 3). **b** Comparison of CDR3β clonotype numbers in tumors from Ctr mRNA or VEGF-C mRNA groups on day 22 after inoculation (*n* = 3). **c** Quantification of CD8^+^ T cells expressing multiple cytokines in tumors from Ctr mRNA or VEGF-C mRNA groups on day 22 after inoculation (*n* = 6). **d** Illustration of the role of MLVs in brain tumors with RT. The MLV-CLN network mediates the RT-modulated anti-tumor immunity and contributes to RT efficacy. Data are presented as means ± SEM. *P < 0.05, **P < 0.01, ***P < 0.001; Student’s *t*-test (**b**, **c**). Data are from at least two **(c**) independent experiments.

We finally analyzed the dynamics of T cell receptor (TCR) CDR3β sequence to explore the change of T cell clonality after VEGF-C mRNA treatment.^43^ Clonal dominance can be visualized by the contribution of the top 50 clones to the repertoire. T cells in VEGF-C mRNA group were more diverse, with the 50 most abundant clones representing only 61.24% of the population, compared with ~92.44% of the total read counts in control mRNA group (Fig. 7a). To further analyze the CDR3β diversity, we compared the numbers of CDR3β clonotypes, which were significantly higher in VEGF-C mRNA group than in control mRNA group (Fig. 7b). In addition, we also compared the frequency of individual V and J genes in these two groups (Supplementary information, Fig. S13a, b). VEGF-C mRNA group exhibited increased usage of mTRBJ1-1, mTRBJ1-6 and mTRBJ2-2, and preferential usage of mTRBV1, mTRBV20, mTRBV24, mTRBV26 and mTRBV29. These data demonstrate that the usage of individual V and J genes distinguishes VEGF-C mRNA treatment group from control group, with a specific gene expression. Besides, the functional CD8^+^ T cells, which produce various cytokines (TNFα, IFNγ, GZMB, and IL2), were increased in the tumor from VEGF-C mRNA group (Fig. 7c). These data indicate an outstanding anti-tumor immune response by the VEGF-C mRNA treatment combined with RT.

## Discussion

The characterization of the MLV-CLN network has opened a new door to the understanding of brain tumor immunology and the mechanisms underlying brain tumor immunotherapy.^20,23^ However, it is unknown whether this network plays a role in the regulation of RT-induced immune responses in brain tumors, while it is known that RT could kill tumor cells directly by causing DNA damage and oxidative stress.^11^ Here, we show that meningeal lymphatics contribute to the inhibitory effects of RT on brain tumors, at least in part, through the modulation of RT-triggered anti-tumor immune responses. The effect of RT on intracranial glioma or metastasized lung cancer was impaired after the excision of CLNs or the disruption of MLVs, as evidenced by a shorter survival time and larger tumor volume in the MLV-disrupted and CLN-excised mice than in control mice. These results are consistent with previous reports that TDLNs are necessary for the efficacy of immunotherapy in peripheral tumors.^44–46^ Taken together, these findings underscore the importance of the MLV-CLN network in regulation of RT-modulated anti-tumor responses and suggest a working model that RT stimulates the trafficking of brain tumor-derived DCs to CLNs and the subsequent activation of CD8^+^ T cells, which rely on intact MLVs (Fig. 7d).

Another important finding in our study was the demonstration that VEGF-C overexpression improved the efficacy of RT in the treatment of brain tumors, likely through enhancing RT-triggered anti-tumor immunity. A recent report has shown that the expression levels of VEGF-C in glioblastoma tissue from patients are lower than those in healthy brain tissue.^23^ In addition, in patients with glioblastoma who are treated with neoadjuvant anti-PD-1, the expression of VEGF-C is strongly correlated with an increase in the infiltration of T cells (as measured by the expression of the T cell marker genes *CD3E*, *CD4*, and *CD8B*) after treatment.^7,23^ In the present study, we further analyzed the transcriptomic profile of gliomas using an available database and found a high correlation of VEGF-C expression among 15 lymphangiogenic factors with CD8^+^ T cell infiltration in human gliomas after RT, suggesting that the ectopic expression of VEGF-C enhances RT-triggered anti-tumor immunity in brain tumors. Indeed, VEGF-C-overexpressing gliomas and metastatic brain tumors from lung cancer were more sensitive to RT than control tumors, very likely mediated through CCL21-dependent DC trafficking and CD8^+^ T cell activation. These results are consistent with our recent findings using a mouse glioma model with immunotherapy.^20^ In addition to DCs, whether MLVs could also play a role in enhancing RT-induced tumor-specific antigen release from irradiated cancer cells needs further investigation.^47^ Consistent with our recent findings,^20^ we demonstrated MLV expansion in mice with VEGF-C-overexpressing gliomas. Glioma-derived VEGF-C in the interstitial fluid may enter the CSF circulation and stimulate MLV to grow during drainage. Therefore, VEGF-C may enhance anti-tumor immune responses by promoting the drainage of tumor antigens via MLV expansion.

A previous study showed that the transfection of a VEGF-C mRNA construct strengthened the efficacy of immunotherapy against glioma.^23^ In our study here, VEGF-C mRNA delivery into the CSF increased the survival time of mice bearing gliomas and reduced tumor volume after RT. Recently, combination immunotherapy strategies with RT in glioma treatment have been investigated in both preclinical and clinical studies.^17,48^ Although the clinical benefit of these combination treatments awaits confirmation, more than 10 clinical trials using immunotherapies combined with RT for gliomas are in progress.^17,48,49^ Together, our results suggest the potential of VEGF-C mRNA as a new means of RT-related combination therapy. Therefore, the present study reveals a new role of meningeal lymphatics in treating brain tumors by RT and RT-triggered anti-tumor immunity and suggests the potential of VEGF-C mRNA in combination therapies with RT for brain tumors.

## Materials and methods

### Reagents and antibodies

Tamoxifen (T5648) and 2,2,2-tribromoethanol (T48402) were from Sigma; collagenase type I (4196) was from Worthington; and collagenase type D (11088866001) was from Roche. RIPA buffer (R0020) was from Solarbio Life Science. FITC-conjugated latex microspheres (17152-10) were from Polysciences and Visudyne was from MedChemExpress (HYB0146). Cell Stimulation Cocktail (plus protein transport inhibitors) (500×; 00-4975-93) were from eBioscience. The cell strainers (70 μm, 258368) were from NEST Biotechnology. The following antibodies were used for OCT-embedded sections: anti-Lyve1 (rabbit, 1:500, Abcam, ab14917), and anti-CCL21 (goat, 1:500, R&D Systems, AF457). Alexa Fluor 488/555/647 donkey anti-rabbit/rat/goat IgG antibodies were from Invitrogen. Anti-VEGF-C antibody (mouse, 1:500, Santa Cruz, sc-374628) was used for western blot analysis. The following antibodies were used for fluorescence-activated cell sorting: anti-CD45-Percp-5.5 (1:300, Invitrogen, 45-0451-82), anti-CD3e-FITC (1:300, Invitrogen, 11-0032-82), anti-CD4-APC (1:300, Invitrogen, 17-0042-82), anti-CD8a-APC (1:300, Invitrogen, 17-0081-82), anti-CD11C-PE (1:300, Invitrogen, 12-0114-82), anti-MHCII-Super Bright 702 (1:300, Invitrogen, 67-5321-82), anti-Foxp3-PE (1:300, Invitrogen, 12-5773-80), anti-Ki67-PE (1:300, Invitrogen, 12-5698-80), anti-CD11b-PE (1:300, BD Bioscience, 557397), anti-F4/80-BV421 (1:300, BD, 565411), anti-IFNγ-PE-CY7 (1:100, Invitrogen, 25-7311-82), anti-Granzyme B-PE-CY7 (1:100, Invitrogen, 25-8898-82), anti-IL2-PE-CY7 (1:50, Invitrogen, 25-7021-82), anti-TNFα-PE (1:100, Invitrogen, 12-7321-81), anti-TCF7-PE (1:100, Cell Signaling Technology, 14456S), rat IgG2b isotype-PE (1:300, Invitrogen, 12-4031-81), rat IgG2b isotype-PE-Cy7 (1:300, Invitrogen, 25-7311-82), and fixable viability stain 510 (1:5000, BD Bioscience, 564406).

### Animals

Male C57BL/6 mice (6–8 weeks old) were used. The Prox1-CreERT2 line (C57BL/6) was a gift from Dr. Taija Makinen (Department of Immunology, Genetics and Pathology, Uppsala University, Sweden). The Prox1-CreERT2 mice were interbred with VEGFR3-flox mice (C57BL/6, generated by Nanjing Biomedical Research Institute of Nanjing University) to generate Prox1-CreERT2;VEGFR3-flox mice. To establish an MLV-deficient transgenic mouse model, Prox-CreRT2^+/–^;VEGFR3-flox^+/+^ mice and their Prox-CreRT2^–/–^;VEGFR3-flox^+/+^ littermates were administered 100 μL of 10 mg/mL tamoxifen by intraperitoneal (i.p.) injection daily for 5 consecutive days, beginning on postnatal day 5. Animals were maintained in the Center for Experimental Animals (a facility accredited by the Association for Assessment and Accreditation of Laboratory Animal Care) at Peking University, Beijing, China. All procedures involving animals followed protocols approved by the Committee for Animal Research of Peking University and conformed to the Guide for the Care and Use of Laboratory Animals. Besides, the maximal tumor size permitted by the Committee for Animal Research of Peking University was 2000 mm^3^.

### Cell lines

293T cells, mouse glioma GL261 cells, and mouse lung carcinoma LLC cells were cultured and maintained in Dulbecco’s modified Eagle’s medium (DMEM) containing 10% fetal bovine serum (FBS). The cells were all cultured at 37 °C in a humidified atmosphere of 5% CO_2_.

### Animal model with striatal injection

Mice aged 7–8 weeks were anesthetized with 2,2,2-tribromoethanol and fixed in a stereotactic frame. After shaving the head, an incision was made and the skull was exposed. One microliter of PBS containing 50,000 GL261 cells or 20,000 LLC cells was stereotaxically injected at 2 mm lateral to bregma at a depth of 3 mm below the dura with a 30-gauge Hamilton syringe. The injection was performed over a 10-min period, then the skin was closed, and the mice were allowed to recover on a heating pad. To perform RT, a lead shield was carefully used to avoid radiation damage of other parts of the mouse.

### LN resection

Deep and superficial CLNs were removed bilaterally from C57 mice 7 days before tumor implantation as previously described.^50^ Briefly, mice were anesthetized with 2,2,2-tribromoethanol and a sagittal incision was made in the midline of the neck. Then, 8 superficial and 2 deep CLNs were removed under a surgical microscope. After surgery, buprenorphine (subcutaneous) and antibiotic (i.p.) were injected. Tumor cells were transplanted after 1 week of recovery.

### Ablation of dorsal MLVs

Mice aged 7–8 weeks were anesthetized with 2,2,2-tribromoethanol. Visudyne was reconstituted following the manufacturer’s instructions (2 mg/mL), and 5 μL was injected into the cisterna magna. After 15 min, Visudyne was photo-converted with a non-thermal 689-nm wavelength laser at different spots through the intact skull as previously reported.^20^

### Establishment of GL261 and LLC cells overexpressing vector or VEGF-C

We constructed retroviral vectors with or without *VEGF-C* gene (vector or VEGF-C group). The retrovirus was made in 293T cells, and the culture supernatant was used to infect LLC and GL261 cells. Puromycin (0.75 mg/L) was used to screen for stable cell pools after infection with retrovirus.

### CCL21 blockade

For CCL21 blocking assays, tumor-bearing mice were given i.p. injections of IgG isotype control antibody (10 μg, MAB006, R&D System) or CCL21-blocking antibody (10 μg, MAB4572, R&D System) on days 15, 17, and 19 after inoculation.

### Generation and delivery of VEGF-C mRNA

VEGF-C mRNA and control mRNA (luciferase mRNA) were prepared by in vitro transcription as previously reported.^51^ Briefly, the Igκ-VEGF-C-Flag sequence was cloned into pEasy vector (TransGen Biotech) under the control of the T7 promoter. Then the Igκ-VEGF-C-Flag construct was linearized by the restriction enzyme *Bgl*II before being used as the DNA template. A HiScribe T7 ARCA mRNA kit (New England Biolabs, E2060S) was used for mRNA generation, mRNA capping, DNA removal, mRNA tailing, and purification. 5 μg of mRNAs complexed to jet-PEI (poluplus) was injected into the CSF via the posterior cistern for in vivo transfection.

### Tumor volume and tumor weight measurement

Tumor volume was measured by MRI. Briefly, we used T2-weighted rapid acquisition with relaxation enhancement (RARE) images to assess tumor volume. The acquisition parameters were as follows: TE = 10, RARE factor = 16, TR = 3000 ms, NA = 4, 11 image slices, 0.5-mm slice thickness, and 150 μm in-plane resolution. Tumor area was determined from the T2 hyperintense regions in the brain as previously reported.^20^ Tumor volume was calculated by length × width^2^ × 0.52. Tumor weight was also measured immediately after the tumor was isolated from the brain.

### Flow cytometry

All flow cytometry gating strategies are shown in Supplementary information, Fig. S1a–f. Briefly, intracranial tumors were incubated in Ca^2+^-phosphate buffered saline (PBS) containing 0.2% type I collagenase at 37 °C for 30 min, and the deep CLNs were collected and incubated in PBS containing 0.1% collagenase D at 37 °C for 1 h. At the end of incubation, the tissue fragments were passed through 70-μm nylon mesh cell strainers after neutralization with 10% FBS in DMEM. After centrifugation, the cells were re-suspended and stained for CD45, CD3e, CD4, CD8, Foxp3, Ki67, CD11c, MHCII, CD11b, F4/80 and TCF7.

To assess DC trafficking, 2 μL of 0.5-μm FITC-conjugated latex microspheres were injected into the tumors; 24 h later, CLNs were harvested, and single-cell suspensions were prepared and analyzed by flow cytometry for CD11c^+^ MHCII^+^ FITC^+^ DCs.

For cytokine stimulation, surface markers were first stained for 30 min. After washing, cells were stimulated in RPMI-1640 with Cell Stimulation Cocktail plus protein transporter inhibitor (eBioscience) for 5 h at 37 °C. Cells were then fixed for 30 min and stained for intracellular antigen: IFNγ, Granzyme B, IL2 and TNFα.

### Immunofluorescent staining

For whole-mount staining, the meninges attached to the skull were fixed in 4% PFA and then separated from the skull cap. Then they were incubated with PBS containing 2% horse serum, 1% bovine serum albumin (BSA), 0.1% Triton X-100, and 0.05% Tween 20 for 1 h at room temperature. After overnight incubation with primary antibody diluted in PBS with 1% BSA and 0.5% Triton X-100 at 4 °C, they were washed 3 times with PBS for 5 min each and incubated with fluorophore-conjugated secondary antibody (all 1:500, diluted in PBS with 1% BSA and 0.5% Triton X-100) at room temperature for 1 h. The meninges were incubated with DAPI medium before capturing images. Finally, we used ImageJ to quantify the results of the meningeal lymphatics, and each assay included at least 6 points.

### TCR sequencing

All RNA samples received by iRepertoire were amplified using a commercially available multiplex primer mix covering the TCR heavy chain (iRepertoire Inc.). Briefly, reverse transcription and subsequent PCR amplification (RT-PCR) was performed using Qiagen OneStep RT-PCR mix (Qiagen). The cDNA was selected and unused primer was removed by SPRIselect bead selection (Beckman Coulter) followed by a second round of amplification performed with a pair of primers that are specific for communal sites engineered onto the 5′ end of the C- and V-primers used during RT-PCR. The final constructed library includes Illumina sequencing adapters and a 6-nucleotide internal barcode associated with the C-gene primer.

Amplified libraries were multiplexed and pooled for sequencing on the Illumina MiSeq platform using either a 500-cycle kit and sequenced as 250 paired end reads, or a 600-cycle kit and sequenced as 300 paired end reads. The output of the immune receptor sequence covers within the first framework region through the beginning of the constant region, CDR3β. Sequencing raw data were analyzed using the previously described iRmap program.^52^ Briefly, sequence reads were de-multiplexed according to barcode sequences at the 5′ end of reads from the constant region. Reads were then trimmed according to their base qualities with a 2-base sliding window. If either quality value in this window is <20, this sequence stretching from the window to 3′ end was trimmed out from the original read. Trimmed paired-end reads were joined together through overlapping alignment with a modified Needleman-Wunsch algorithm. If paired forward and reverse reads in the overlapping region were not perfectly matched, both forward and reverse reads were thrown out without further consideration. The merged reads were mapped using a Smith-Waterman algorithm to germline V and J reference sequences using an IMGT reference library. To define CDR3β region, the position of CDR3β boundaries of reference sequences from the IMGT database was migrated onto reads through mapping results, and the resulting CDR3β regions were extracted and translated into amino acids.

### Statistical analysis

All experiments were repeated at least twice. The number of animals is specified in each figure legend. Data are expressed as means ± SEM. Statistical analyses were performed by Student’s *t*-test, one-way ANOVA, and the log-rank (Mantel–Cox) test. GraphPad software was used for data analysis. Statistical significance is indicated as follows: *P < 0.05, ** *P* < 0.01, ***P < 0.001.

## Supplementary information

Supplementary information, Fig. S1

Supplementary information, Fig. S2

Supplementary information, Fig. S3

Supplementary information, Fig. S4

Supplementary information, Fig. S5

Supplementary information, Fig. S6

Supplementary information, Fig. S7

Supplementary information, Fig. S8

Supplementary information, Fig. S9

Supplementary information, Fig. S10

Supplementary information, Fig. S11

Supplementary information, Fig. S12

Supplementary information, Fig. S13
