# Supplementary material for: Meningeal lymphatics regulate radiotherapy efficacy through modulating anti-tumor immunity
Source: Cell Res. 2022 Mar 17;32(6):543–54. doi: 10.1038/s41422-022-00639-5 (PMC9159979; doi:10.1038/s41422-022-00639-5)
Supplement: Supplementary file 1 — Supplementary information, Fig. S1 [file 41422_2022_639_MOESM1_ESM.pdf]

Supplementary information, Figure S1

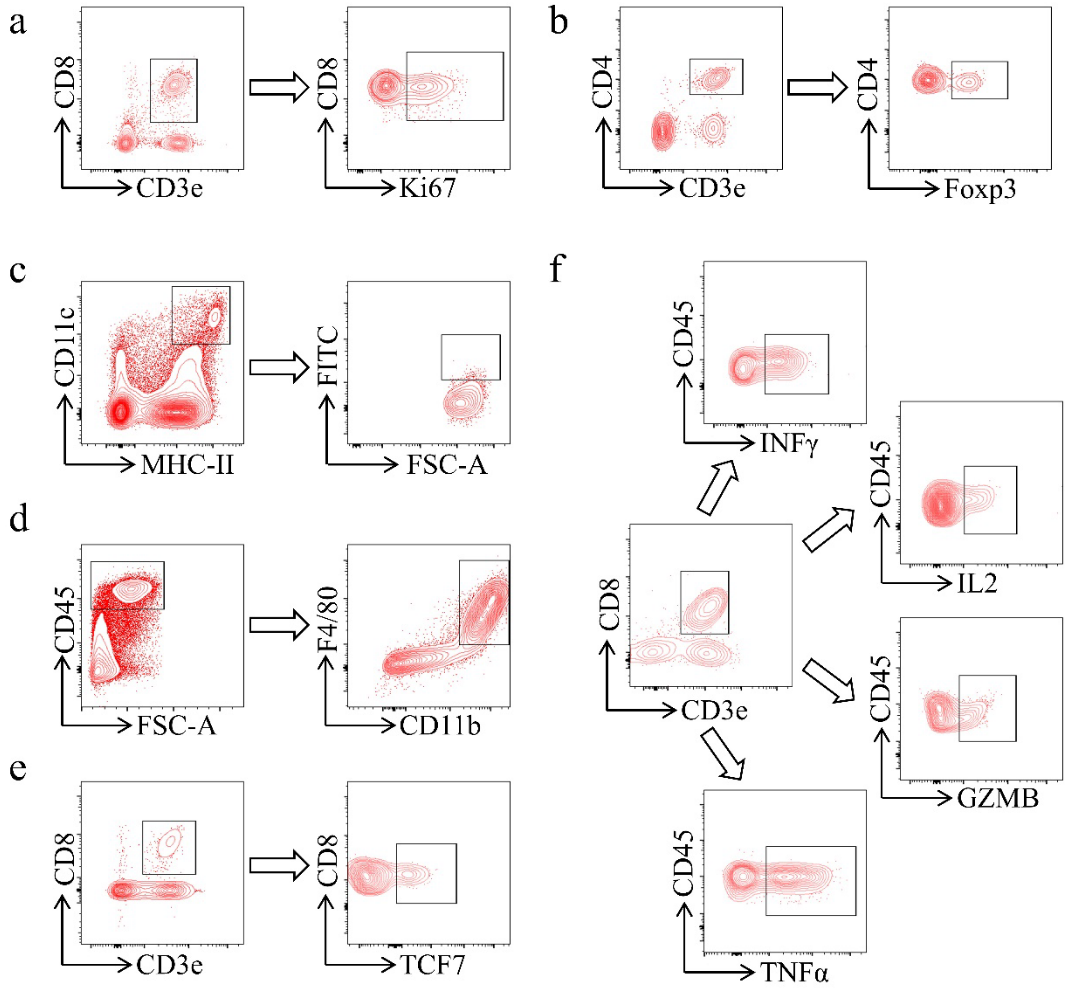

Supplementary information, Figure S1. Flow cytometry gating strategies. Representative contour plots for CD8⁺ Ki67⁺ T cells (a), CD4⁺ Foxp3⁺ T cells (b), CD11c⁺ MHCII⁺ FITC⁺ cells (c; Beads⁺ DCs), CD11b⁺ F4/80⁺ cells (d; tumor associated macrophages), CD8⁺ TCF7⁺ T cells (e), and cytokine production in CD8⁺ T cells (f).
