# Supplementary material for: Meningeal lymphatics regulate radiotherapy efficacy through modulating anti-tumor immunity
Source: Cell Res. 2022 Mar 17;32(6):543–54. doi: 10.1038/s41422-022-00639-5 (PMC9159979; doi:10.1038/s41422-022-00639-5)
Supplement: Supplementary file 2 — Supplementary information, Fig. S2 [file 41422_2022_639_MOESM2_ESM.pdf]

Supplementary information, Figure S2

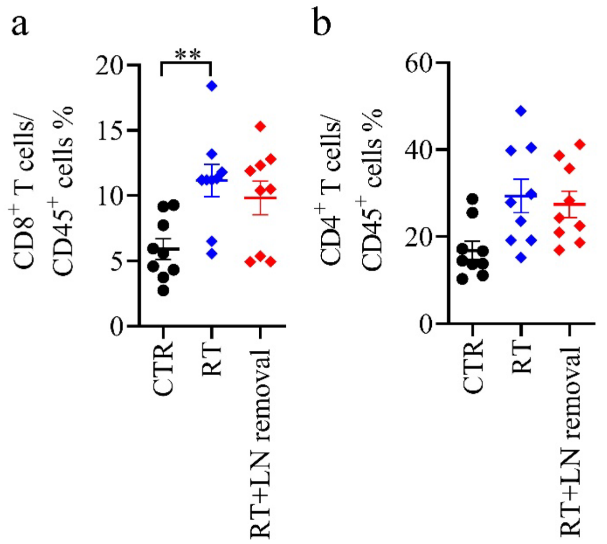

Supplementary information, Figure S2. T cell population in LN removal model. $CD8^+$ T cells (a), and $CD4^+$ T cells (b) in in tumors from CTR, RT, and RT+LN removal groups as percentages of overall $CD45^+$ cells on day 22 after inoculation (n = 9). Data are presented as means ± SEM. **P <0.01; one-way ANOVA (a–b). Data are from at least three (a–b) independent experiments.
