# Supplementary material for: Meningeal lymphatics regulate radiotherapy efficacy through modulating anti-tumor immunity
Source: Cell Res. 2022 Mar 17;32(6):543–54. doi: 10.1038/s41422-022-00639-5 (PMC9159979; doi:10.1038/s41422-022-00639-5)
Supplement: Supplementary file 3 — Supplementary information, Fig. S3 [file 41422_2022_639_MOESM3_ESM.pdf]

Supplementary information, Figure S3

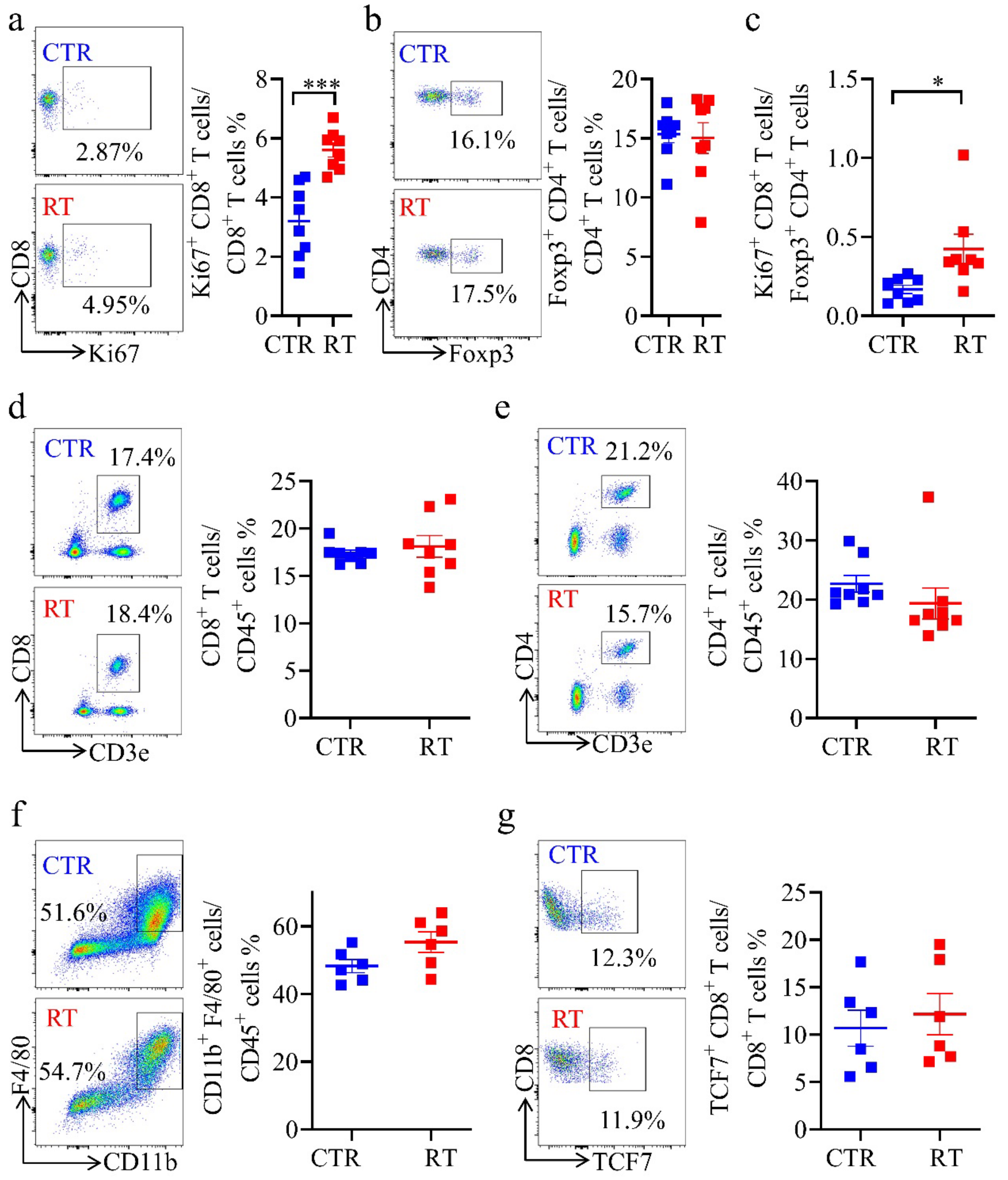

Supplementary information, Figure S3. RT promotes anti-tumor immunity. Representative flow cytometry plots and quantification of CD8$^+$ Ki67$^+$ T cells as percentages of overall CD8$^+$ T cells (a), and CD4$^+$ Foxp3$^+$ T cells as percentages of overall CD4$^+$ T cells (b) in CLNs from GL261 tumor mice with or without RT on day 22 after inoculation (n = 8). c, Ratios of CD8$^+$ Ki67$^+$ T cells to CD4$^+$ Foxp3$^+$ T cells in CLNs from GL261 tumor mice with or without RT (n = 8). d–e, Representative flow cytometry plots and quantification of CD8$^+$ T cells (d), and CD4$^+$ T cells (e) in CLNs

from GL261 tumor mice with or without RT as percentages of overall $CD45^+$ cells on day 22 after inoculation (n = 8). f, Representative flow cytometry plots and quantification of $CD11b^+$ $F4/80^+$ cells in tumors from GL261 tumor mice with or without RT as percentages of overall $CD45^+$ cells on day 22 after inoculation (n = 6). g, Representative flow cytometry plots and quantification of $CD8^+$ $TCF7^+$ T cells in tumors from GL261 tumor mice with or without RT as percentages of overall $CD45^+$ cells on day 22 after inoculation (n = 6). Data are presented as means ± SEM. *P <0.05, ***P <0.001; Student's t test (a–g). Data are from at least three (a–g) independent experiments.
