# Supplementary material for: Meningeal lymphatics regulate radiotherapy efficacy through modulating anti-tumor immunity
Source: Cell Res. 2022 Mar 17;32(6):543–54. doi: 10.1038/s41422-022-00639-5 (PMC9159979; doi:10.1038/s41422-022-00639-5)
Supplement: Supplementary file 4 — Supplementary information, Fig. S4 [file 41422_2022_639_MOESM4_ESM.pdf]

Supplementary information, Figure S4

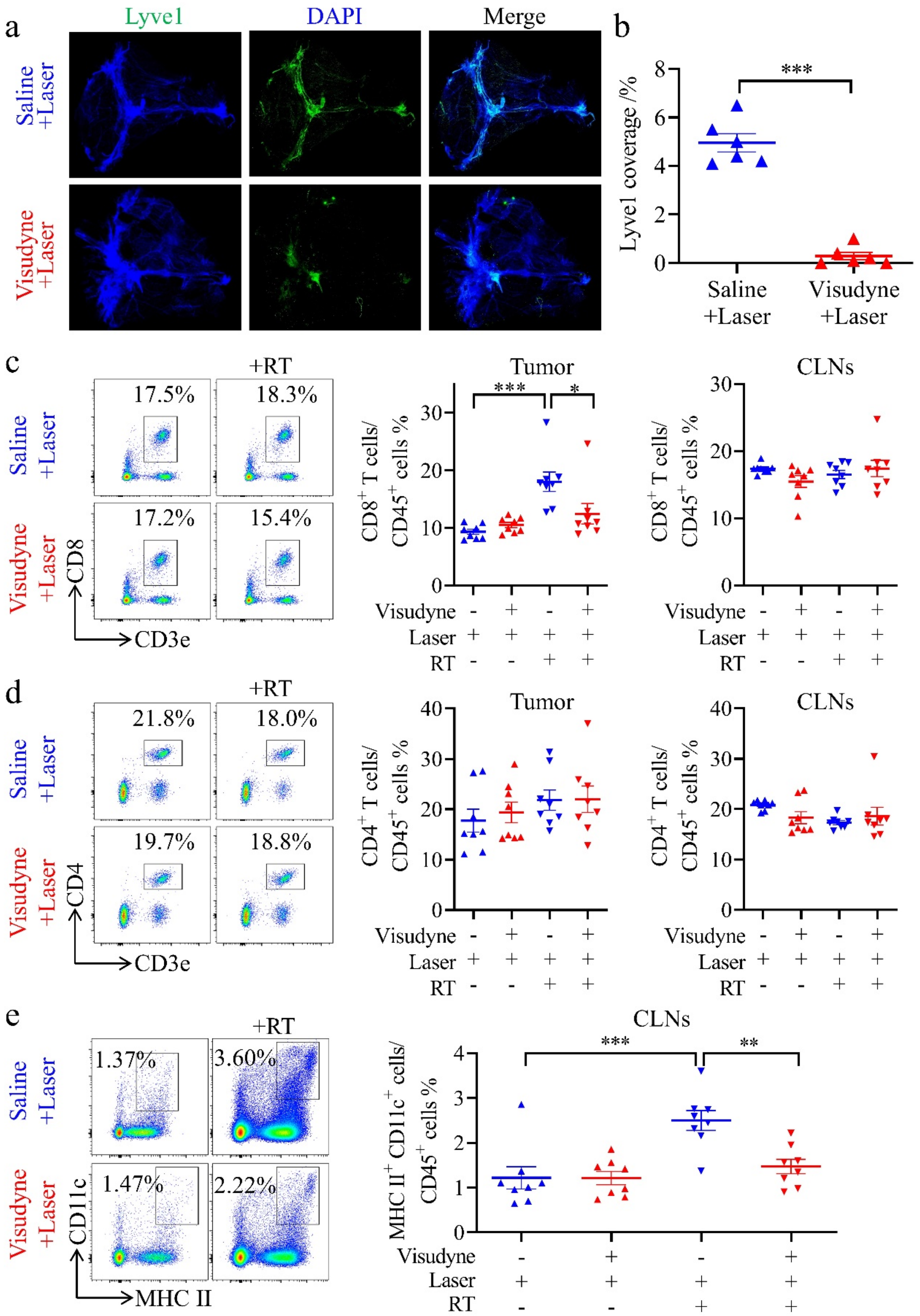

Supplementary information, Figure S4. Establishment of MLV-defective mouse model by Visudyne with laser. a, Lyve1 staining of MLVs in mice treated with laser, with or without Visudyne. b, Percentage area of Lyve1. c–d, Representative flow

cytometry plots of $CD8^+$ T cells (c), and $CD4^+$ T cells (d) in CLNs (left) and quantification (right) in tumors and CLNs from the above groups as percentages of overall $CD45^+$ cells on day 22 after inoculation (n = 8). e, Representative flow cytometry plots and quantification of $CD11c^+$ $MHCII^+$ cells in CLNs from the above groups as percentages of overall $CD45^+$ cells on day 22 after inoculation (n = 8). Data are presented as means ± SEM. *P <0.05, **P <0.01, ***P <0.001; Student's t test (b), one-way ANOVA (c–e). Data are from at least two (a, b) or three (c–e) independent experiments.
