# Supplementary material for: Meningeal lymphatics regulate radiotherapy efficacy through modulating anti-tumor immunity
Source: Cell Res. 2022 Mar 17;32(6):543–54. doi: 10.1038/s41422-022-00639-5 (PMC9159979; doi:10.1038/s41422-022-00639-5)
Supplement: Supplementary file 5 — Supplementary information, Fig. S5 [file 41422_2022_639_MOESM5_ESM.pdf]

Supplementary information, Figure S5

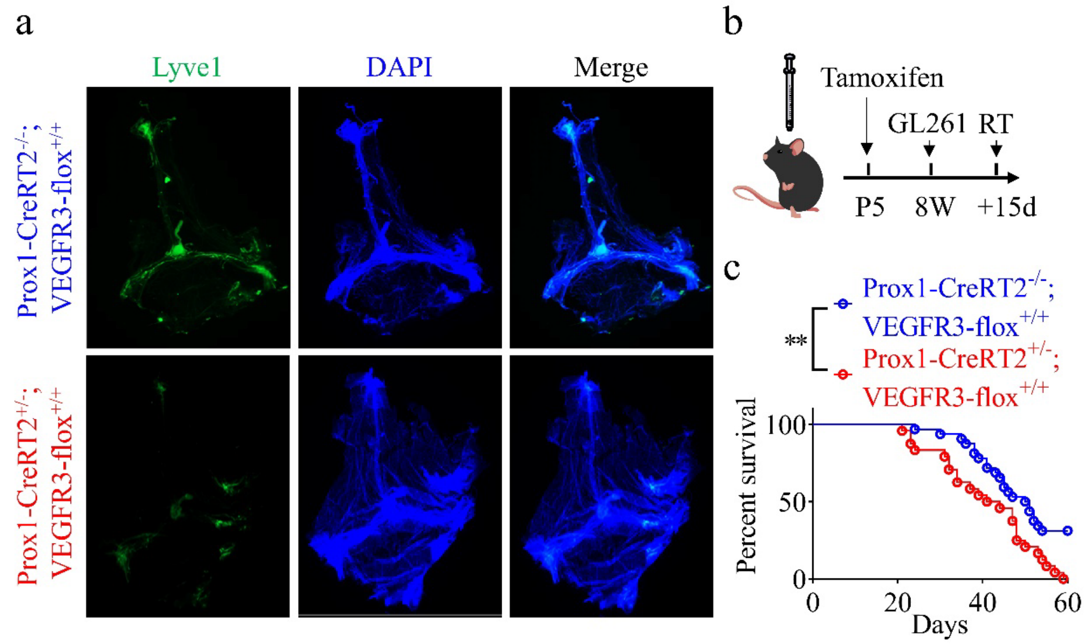

Supplementary information, Figure S5. VEGFR3 knockout in MLVs weakens the therapeutic effect of RT. a, Lyve1 staining of MLVs from Prox1-CreRT2$^{+/-}$; VEGFR3-flox$^{+/+}$ and Prox1-CreRT2$^{-/-}$; VEGFR3-flox$^{+/+}$ mice with tamoxifen treatment. b, Monitoring and treatment scheme. Tamoxifen was injected i.p. on P5 in order to induce VEGFR3 ablation in MLVs. c, Survival of mice with striatal GL261 tumor injection treated with RT (Prox1-CreRT2$^{+/-}$; VEGFR3-flox$^{+/+}$, n =32; Prox1-CreRT2$^{-/-}$; VEGFR3-flox$^{+/+}$, n =24). **P <0.01; log-rank (Mantel–Cox) test (c). Data are from at least two (a, b) independent experiments.
