# Supplementary material for: Meningeal lymphatics regulate radiotherapy efficacy through modulating anti-tumor immunity
Source: Cell Res. 2022 Mar 17;32(6):543–54. doi: 10.1038/s41422-022-00639-5 (PMC9159979; doi:10.1038/s41422-022-00639-5)
Supplement: Supplementary file 6 — Supplementary information, Fig. S6 [file 41422_2022_639_MOESM6_ESM.pdf]

Supplementary information, Figure S6

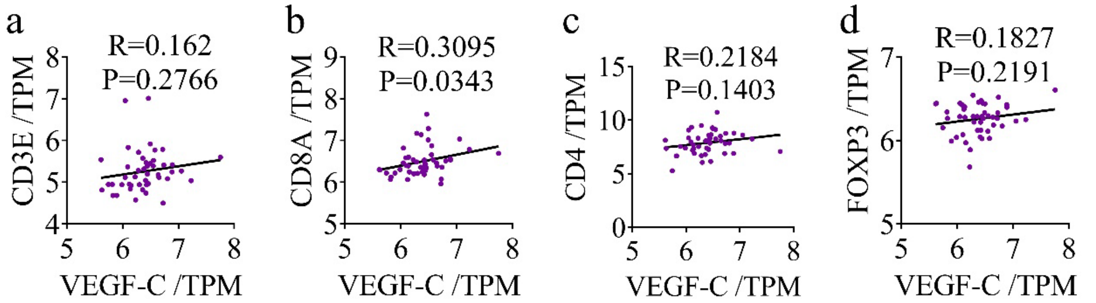

Supplementary information, Figure S6. VEGF-C is closely correlated with anti-tumor immunity in RT from the Gene Expression Omnibus. a–d, Correlation of the expression (transcripts per million; TPM) of VEGF-C and the T cell marker CD3E (a), the CD8+ T cell marker CD8A (b), and the Treg markers CD4 (c) and Foxp3 (d) in glioma patients treated with RT (data from GSE42669 of the Gene Expression Omnibus database; n = 47). P values are Pearson's correlations.
