# Supplementary material for: Meningeal lymphatics regulate radiotherapy efficacy through modulating anti-tumor immunity
Source: Cell Res. 2022 Mar 17;32(6):543–54. doi: 10.1038/s41422-022-00639-5 (PMC9159979; doi:10.1038/s41422-022-00639-5)
Supplement: Supplementary file 7 — Supplementary information, Fig. S7 [file 41422_2022_639_MOESM7_ESM.pdf]

Supplementary information, Figure S7

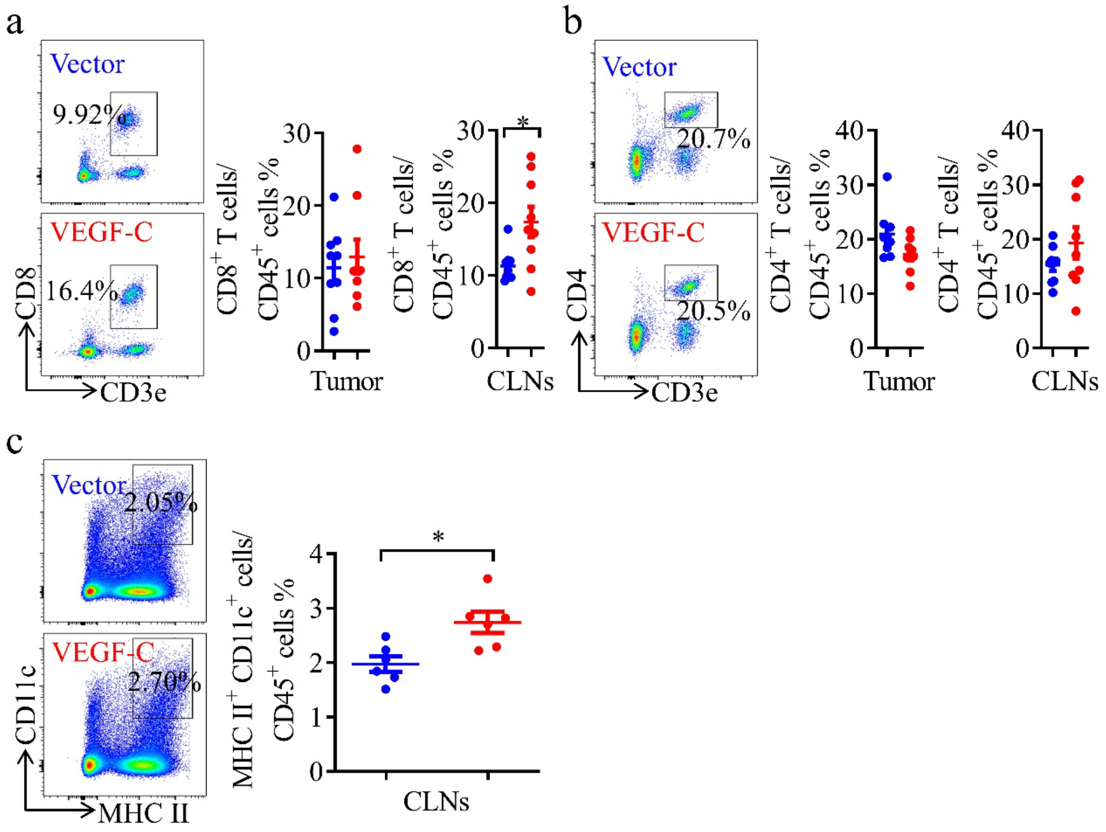

Supplementary information, Figure S7. T cell and DC population in VEGF-C-overexpressing gliomas. a–b, Representative flow cytometry plots of $CD8^+$ T cells (a), and $CD4^+$ T cells (b) in CLNs (left) and quantification (right) in tumors and CLNs from mice with striatal Vector-GL261 or VEGF-C-GL261 tumor injection treated with RT as percentages of overall $CD45^+$ cells on day 22 after inoculation (n = 9). c, Representative flow cytometry plots and quantification of $CD11c^+$ $MHCII^+$ cells in CLNs from mice with striatal Vector-GL261 or VEGF-C-GL261 tumor injection treated with RT as percentages of overall $CD45^+$ cells on day 22 after inoculation (n = 6). Data are presented as means ± SEM. *P <0.05; Student's t test (a–c). Data are from at least three (a–c) independent experiments.
