# Supplementary material for: Meningeal lymphatics regulate radiotherapy efficacy through modulating anti-tumor immunity
Source: Cell Res. 2022 Mar 17;32(6):543–54. doi: 10.1038/s41422-022-00639-5 (PMC9159979; doi:10.1038/s41422-022-00639-5)
Supplement: Supplementary file 8 — Supplementary information, Fig. S8 [file 41422_2022_639_MOESM8_ESM.pdf]

Supplementary information, Figure S8

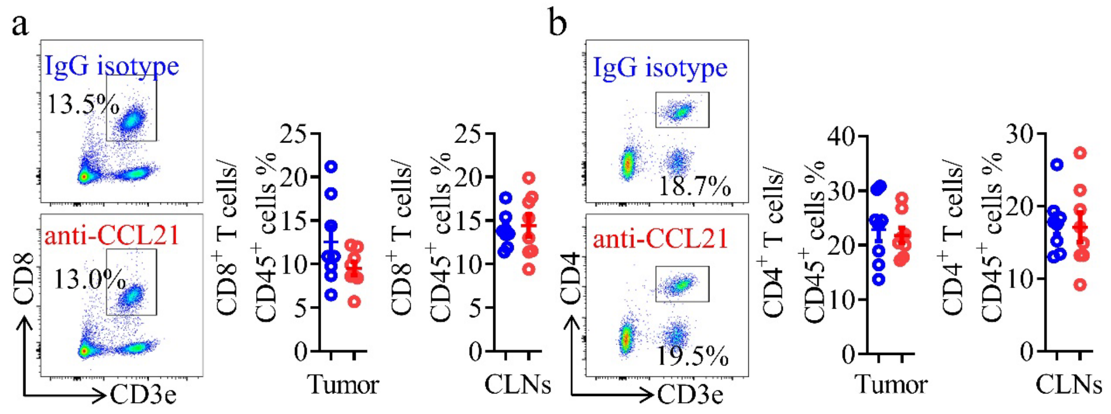

Supplementary information, Figure S8. T cell population after CCL21 blockade. a–b, Representative flow cytometry plots of CD8$^+$ T cells (a), and CD4$^+$ T cells (b) in CLNs (left) and quantification (right) in tumors and CLNs from IgG isotype or anti-CCL21 groups as percentages of overall CD45$^+$ cells on day 22 after inoculation (n = 8). Data are presented as means ± SEM. Student's t test (a–b). Data are from at least three (a–b) independent experiments.
