# Supplementary material for: Meningeal lymphatics regulate radiotherapy efficacy through modulating anti-tumor immunity
Source: Cell Res. 2022 Mar 17;32(6):543–54. doi: 10.1038/s41422-022-00639-5 (PMC9159979; doi:10.1038/s41422-022-00639-5)
Supplement: Supplementary file 9 — Supplementary information, Fig. S9 [file 41422_2022_639_MOESM9_ESM.pdf]

Supplementary information, Figure S9

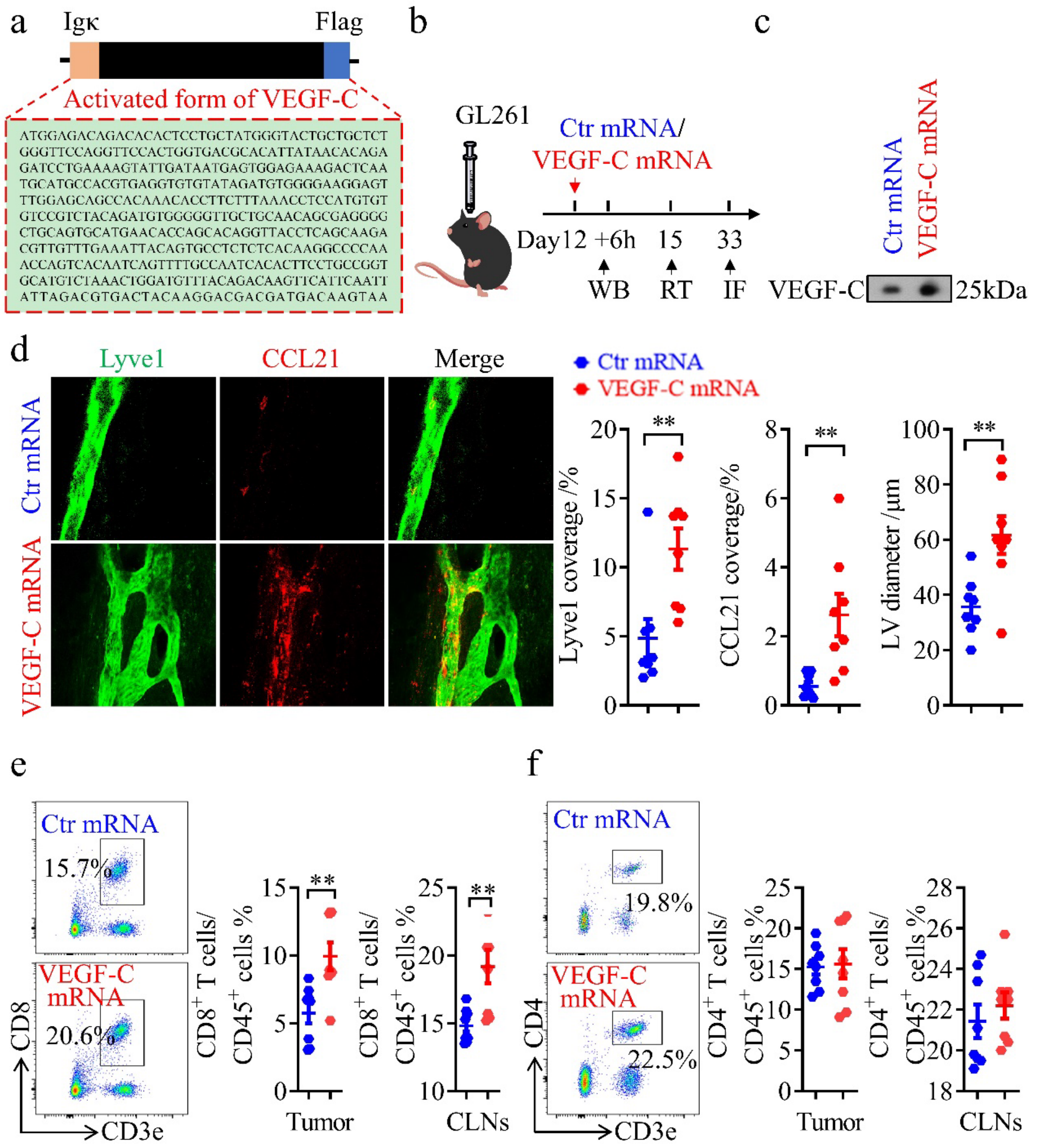

Supplementary information, Figure S9. VEGF-C mRNA induces VEGF-C overexpression. a, Construction of Igκ-VEGF-C-flag mRNA. b, Monitoring and treatment scheme. Control (Ctr) mRNA or VEGF-C mRNA was injected on day 12 after inoculation. c, Immunoprecipitation of secreted VEGF-C protein in cerebrospinal fluid 6 h after mRNA injection. d, Left panels, Lyve1 and CCL21 staining of MLVs in mice treated with Ctr mRNA or VEGF-C mRNA, 21 days after mRNA injection. Right panels, quantification of the percentage area of Lyve1 or CCL21, and the LV diameter (n = 8). e–f, Representative flow cytometry plots of $CD8^+$ T cells (e), and $CD4^+$ T cells

(f) in CLNs (left) and quantification (right) in tumors and CLNs from Ctr mRNA or VEGF-C mRNA groups as percentages of overall $CD45^+$ cells on day 22 after inoculation (n = 8). Data are presented as means ± SEM. **P <0.01; Student's t test (d–f). Data are from at least two (c–f) independent experiments.
