# Supplementary material for: Meningeal lymphatics regulate radiotherapy efficacy through modulating anti-tumor immunity
Source: Cell Res. 2022 Mar 17;32(6):543–54. doi: 10.1038/s41422-022-00639-5 (PMC9159979; doi:10.1038/s41422-022-00639-5)
Supplement: Supplementary file 10 — Supplementary information, Fig. S10 [file 41422_2022_639_MOESM10_ESM.pdf]

Supplementary information, Figure S10

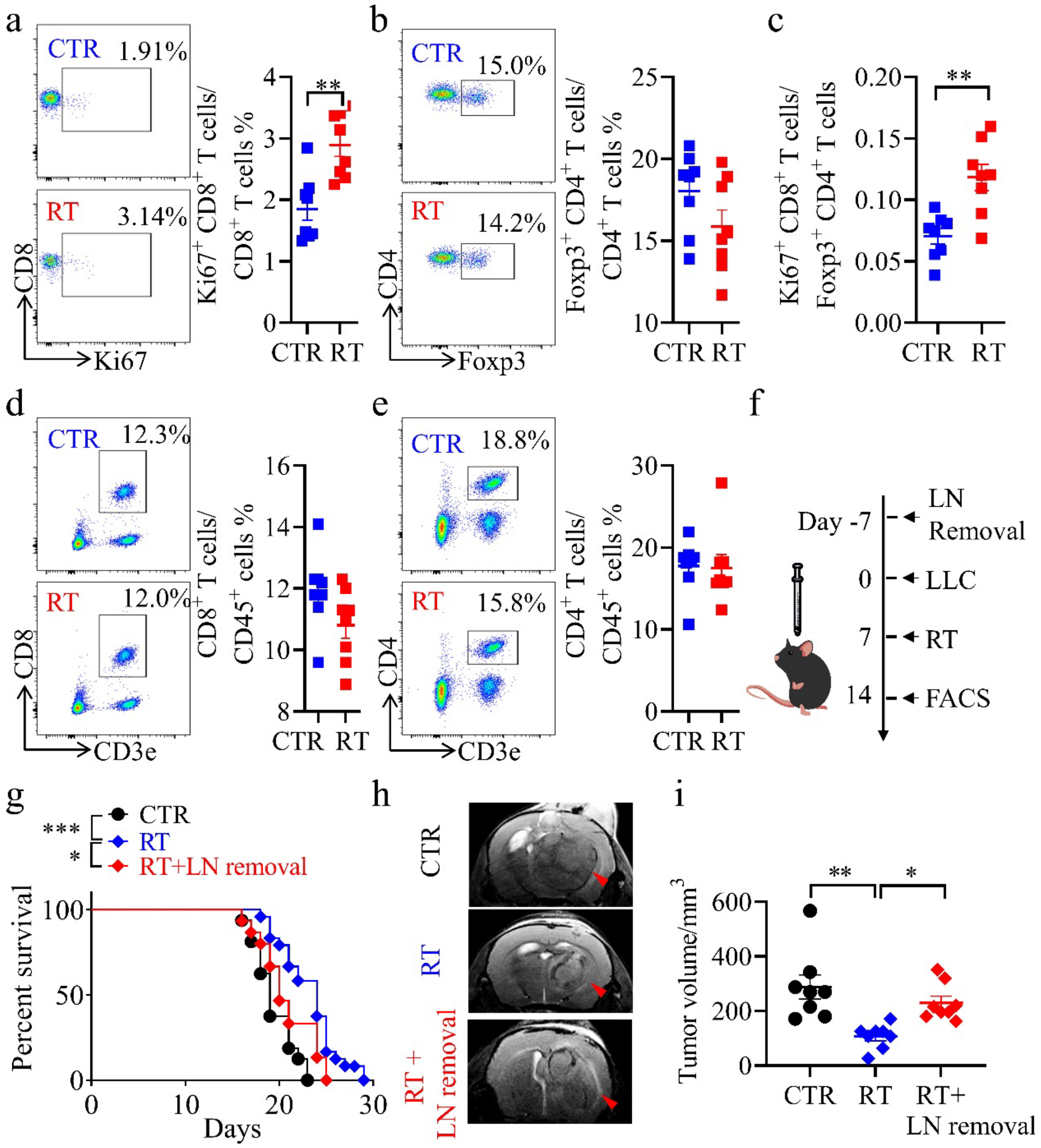

Supplementary information, Figure S10. RT promotes anti-tumor immunity and LN removal weakens the therapeutic effect of RT in a model of metastatic lung cancer. a–b, Representative flow cytometry plots of CD8$^+$ Ki67$^+$ T cells as percentages of overall CD8$^+$ T cells (a), and CD4$^+$ Foxp3$^+$ T cells as percentages of overall CD4$^+$ T cells (b) in CLNs from mice with LLC tumors with or without RT (left) and quantification (right) in CLNs on day 14 after inoculation (n = 8). c, Ratios of CD8$^+$ Ki67$^+$ T cells to CD4$^+$ Foxp3$^+$ T cells in CLNs from mice with LLC tumors with or without RT (n = 8). d–e, Representative flow cytometry plots and quantification of CD8$^+$ T cells (d), and CD4$^+$ T cells (e) in CLNs from mice with LLC tumors with or

without RT in CLNs as percentages of overall $CD45^+$ cells on day 14 after inoculation (n = 8). f, Schematic of the mouse LLC tumor model with RT. g, Survival of mice with striatal LLC tumor injection, without treatment (CTR, n = 16), treated with RT alone (RT, n = 24), or treated with RT plus LN removal (RT+LN removal, n = 15). h, Representative T2-weighted single brain slices from the CTR, RT, and RT+LN removal groups (triangles indicate tumors). i, Tumor volume in the CTR, RT, and RT+LN removal groups (n = 8). Data are presented as means ± SEM. *P <0.05, **P <0.01, ***P <0.001; Student's t test (a–e); log-rank (Mantel–Cox) test (g); one-way ANOVA (I). Data are from at least three (a–e, g) or two (h, i) independent experiments.
