# Supplementary material for: Meningeal lymphatics regulate radiotherapy efficacy through modulating anti-tumor immunity
Source: Cell Res. 2022 Mar 17;32(6):543–54. doi: 10.1038/s41422-022-00639-5 (PMC9159979; doi:10.1038/s41422-022-00639-5)
Supplement: Supplementary file 11 — Supplementary information, Fig. S11 [file 41422_2022_639_MOESM11_ESM.pdf]

Supplementary information, Figure S11

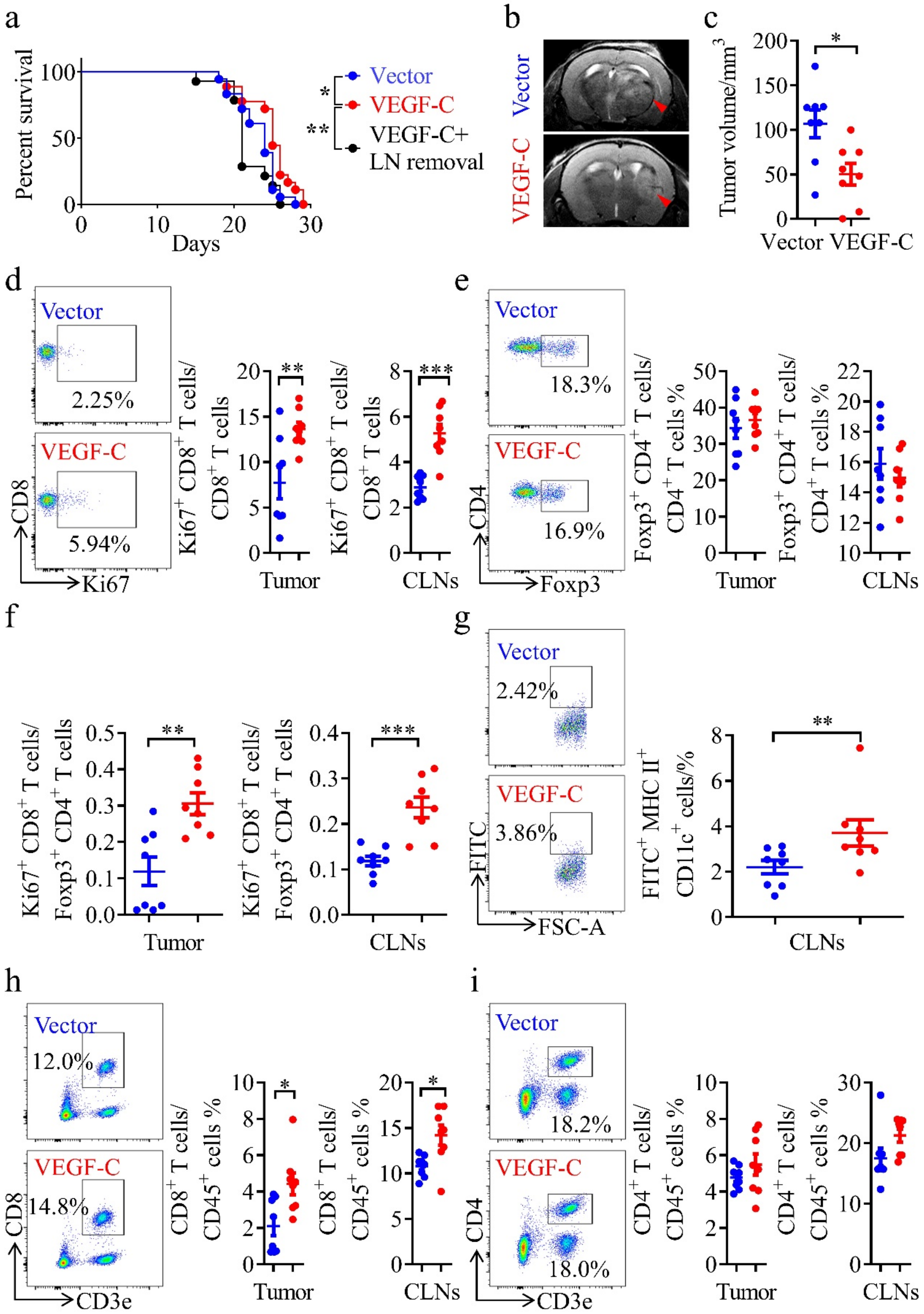

Supplementary information, Figure S11. Tumor-derived VEGF-C improves the therapeutic effect of RT in a model of metastatic lung cancer. a, Survival of mice with striatal Vector-LLC or VEGF-C-LLC tumor injection treated with RT (Vector, n =

18; VEGF-C, n = 18; VEGF-C + LN removal, n = 14). b, Representative T2-weighted single brain slices from mice with striatal Vector-LLC or VEGF-C-LLC tumor injection treated with RT (triangles indicate tumors). c, Tumor volume in mice with striatal Vector-LLC or VEGF-C-LLC tumor injection treated with RT (n = 8). d–e, Representative flow cytometry plots of $CD8^+$ $Ki67^+$ T cells as percentages of overall $CD8^+$ T cells (d), and $CD4^+$ $Foxp3^+$ T cells as percentages of overall $CD4^+$ T cells (e) in CLNs (left) and quantification (right) in tumors and CLNs from mice with striatal Vector-LLC or VEGF-C-LLC tumor injection treated with RT on day 14 after inoculation (n = 8). f, Ratios of $CD8^+$ $Ki67^+$ T cells to $CD4^+$ $Foxp3^+$ T cells in tumors and CLNs from mice with striatal Vector-LLC or VEGF-C-LLC tumor injection treated with RT (n = 8). g, Left panel, representative flow cytometry dot plots of DC trafficking from LLC tumors to CLNs of mice with striatal Vector-LLC or VEGF-C-LLC tumor injection treated with RT by the quantity of $FITC^+$ DCs in the CLNs 24 h after intratumoral injection of FITC-labeled latex beads. Right panel, quantification of $Bead^+$ DCs in the CLNs of mice with striatal Vector-LLC or VEGF-C-LLC tumor injection treated with RT (n = 8). h–i, Representative flow cytometry plots of $CD8^+$ T cells (h), and $CD4^+$ T cells (i) in CLNs (left) and quantification (right) in tumors and CLNs from mice with striatal Vector-LLC or VEGF-C-LLC tumor injection treated with RT as percentages of overall $CD45^+$ cells on day 14 after inoculation (n = 8). Data are presented as means ± SEM. *P <0.05, **P <0.01, ***P <0.001; log-rank (Mantel–Cox) test (a); Student' t test (c–i). Data are from at least three (a, d–i) or two (b, c) independent experiments.
