# Supplementary material for: Meningeal lymphatics regulate radiotherapy efficacy through modulating anti-tumor immunity
Source: Cell Res. 2022 Mar 17;32(6):543–54. doi: 10.1038/s41422-022-00639-5 (PMC9159979; doi:10.1038/s41422-022-00639-5)
Supplement: Supplementary file 12 — Supplementary information, Fig. S12 [file 41422_2022_639_MOESM12_ESM.pdf]

Supplementary information, Figure S12

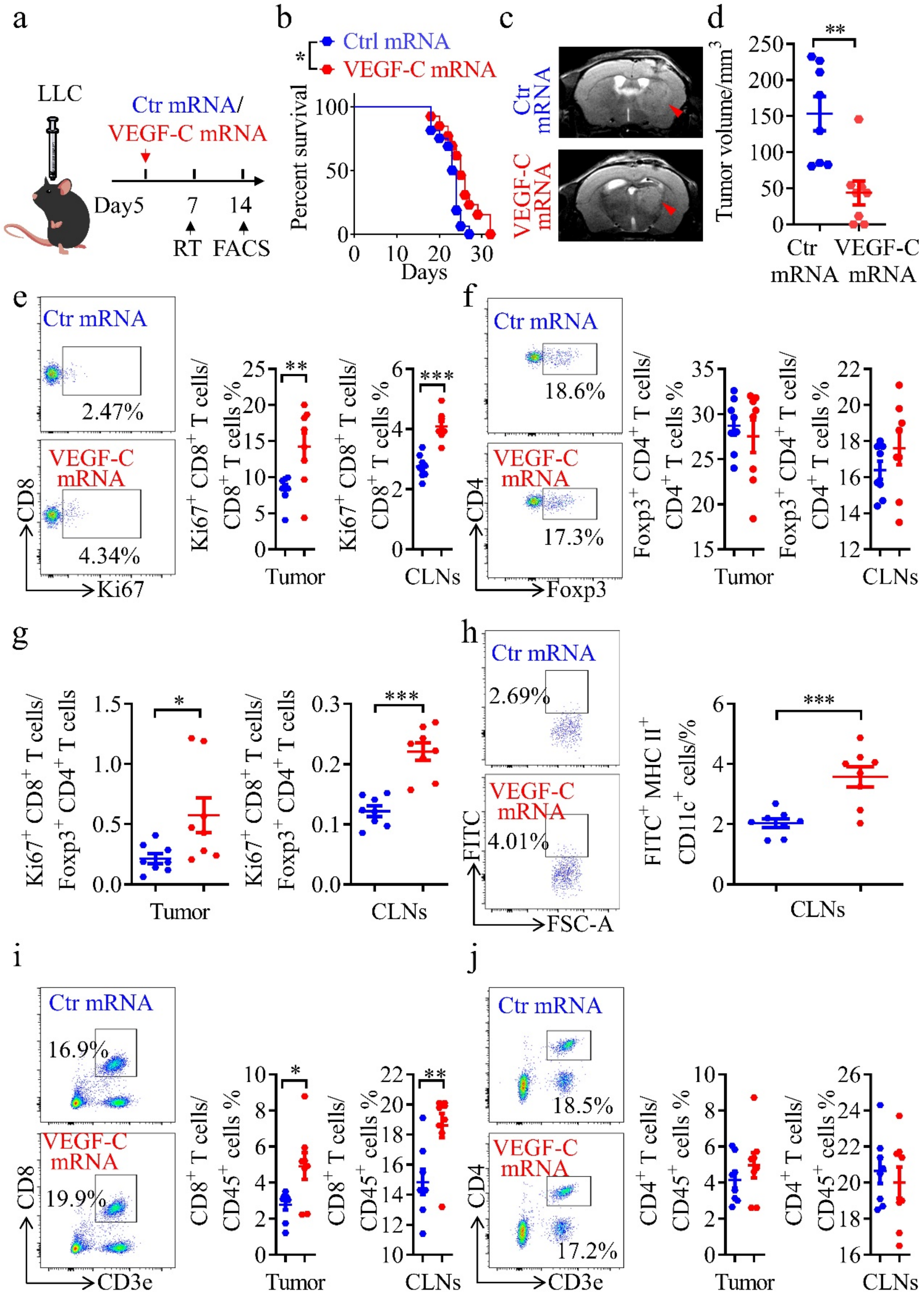

Supplementary information, Figure S12. VEGF-C mRNA also enhances the efficiency of RT in a model of metastatic lung cancer. a, Monitoring and treatment scheme. mRNA was injected on day 5 after inoculation. b, Survival of mice with

striatal LLC tumor injection treated with RT, and with or without VEGF-C mRNA (Ctr mRNA, n = 13; VEGF-C mRNA, n = 16). c, Representative T2-weighted single brain slices of mice from Ctr mRNA or VEGF-C mRNA groups (triangles indicate tumors). d, Tumor volume in mice from Ctr mRNA or VEGF-C mRNA groups (n = 8). e–f, Representative flow cytometry plots of $CD8^+$ $Ki67^+$ T cells as percentages of overall $CD8^+$ T cells (e), and $CD4^+$ $Foxp3^+$ T cells as percentages of overall $CD4^+$ T cells (f) in CLNs (left) and quantification (right) in tumors and CLNs from Ctr mRNA or VEGF-C mRNA groups on day 14 after inoculation (n = 8). g, Ratios of $CD8^+$ $Ki67^+$ T cells to $CD4^+$ $Foxp3^+$ T cells in tumors and CLNs from Ctr mRNA or VEGF-C mRNA groups (n = 8). h, Left panel, representative flow cytometry dot plots of DC trafficking from LLC tumors to CLNs of mice in the above groups by the quantity of $CD11c^+$ $MHCII^+$ $FITC^+$ cells in the CLNs 24 h after intratumoral injection of FITC-labeled latex beads. Right panel, quantification of $FITC^+$ DCs in the CLNs of the Ctr mRNA or VEGF-C mRNA groups (n = 8). i–j, Representative flow cytometry plots of $CD8^+$ T cells (i), and $CD4^+$ T cells (j) in CLNs (left) and quantification (right) in tumors and CLNs from Ctr mRNA or VEGF-C mRNA groups as percentages of overall $CD45^+$ cells on day 14 after inoculation (n = 8). Data are presented as means ± SEM. *P <0.05, **P <0.01, ***P <0.001; log-rank (Mantel–Cox) test (b); Student's t test (d–j). Data are from at least three (b, e–j) or two (c, d) independent experiments.
