# Supplementary material for: Meningeal lymphatics regulate radiotherapy efficacy through modulating anti-tumor immunity
Source: Cell Res. 2022 Mar 17;32(6):543–54. doi: 10.1038/s41422-022-00639-5 (PMC9159979; doi:10.1038/s41422-022-00639-5)
Supplement: Supplementary file 13 — Supplementary information, Fig. S13 [file 41422_2022_639_MOESM13_ESM.pdf]

Supplementary information, Figure S13

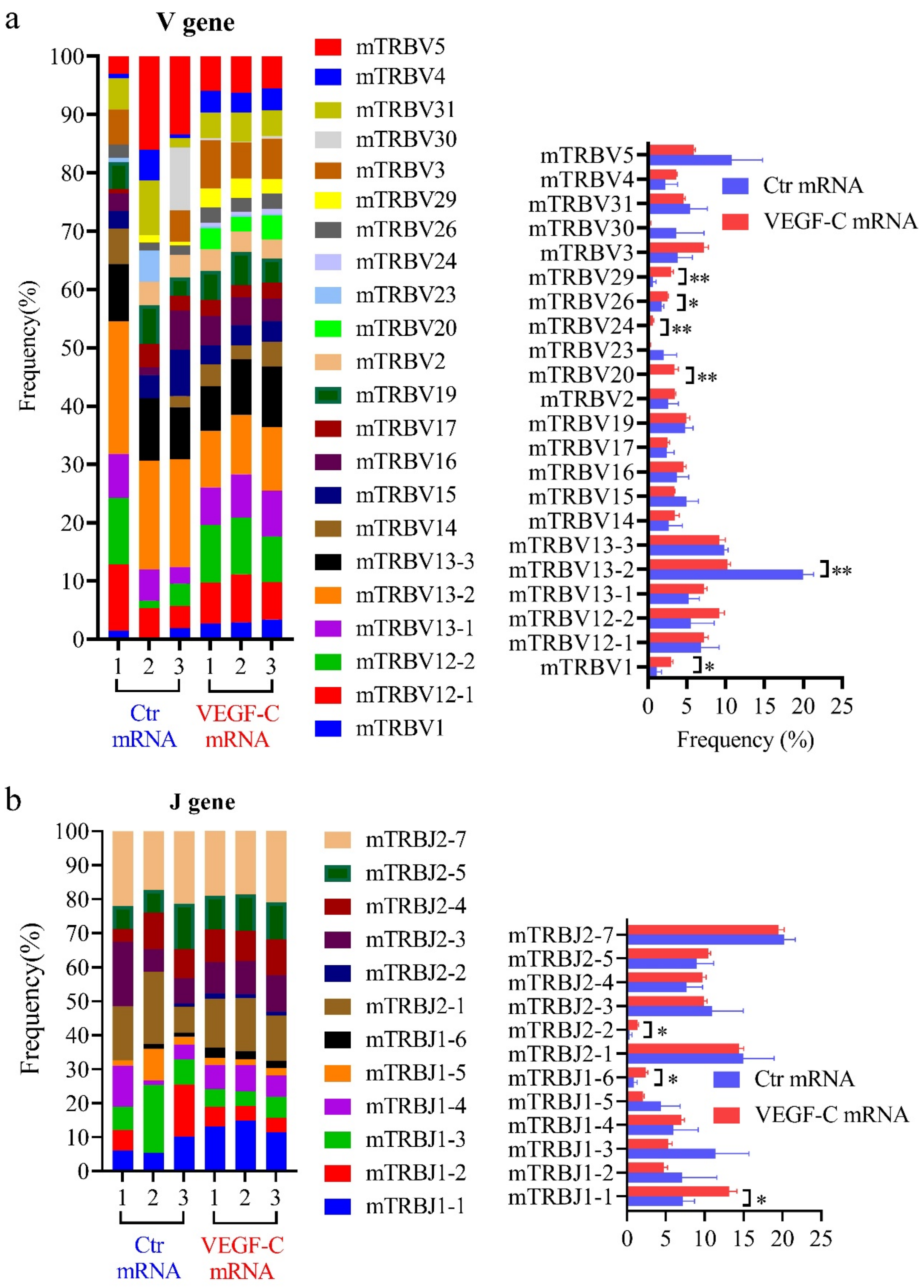

Supplementary information, Figure S13. The gene expression of the V and J gene segments in glioma after RT. a, Left panel, the gene expression of the V gene segments in tumor from Ctr mRNA or VEGF-C mRNA groups on day 22 after

inoculation (n = 3); Right panel, frequency of V gene segments in tumor from Ctr mRNA or VEGF-C mRNA groups on day 22 after inoculation (n = 3). b, Left panel, the gene expression of the J gene segments in tumor from Ctr mRNA or VEGF-C mRNA groups on day 22 after inoculation (n = 3); Right panel, frequency of J gene segments in tumor from Ctr mRNA or VEGF-C mRNA groups on day 22 after inoculation (n = 3). Data are presented as means ± SEM. *P <0.05, **P <0.01; Student's t test (a–b).
